# Hyperandrogenism and insulin resistance contribute to hepatic steatosis and inflammation in female rat liver

**DOI:** 10.18632/oncotarget.24477

**Published:** 2018-02-09

**Authors:** Yuehui Zhang, Fanci Meng, Xiaoyan Sun, Xue Sun, Min Hu, Peng Cui, Edvin Vestin, Xin Li, Wei Li, Xiao-Ke Wu, John-Olov Jansson, Linus R. Shao, Håkan Billig

**Affiliations:** ^1^ Department of Obstetrics and Gynecology, Key Laboratory and Unit of Infertility in Chinese Medicine, First Affiliated Hospital, Heilongjiang University of Chinese Medicine, 150040 Harbin, China; ^2^ Department of Physiology/Endocrinology, Institute of Neuroscience and Physiology, The Sahlgrenska Academy, University of Gothenburg, 40530 Gothenburg, Sweden; ^3^ Department of Integrative Medicine and Neurobiology, State Key Lab of Medical Neurobiology, Shanghai Medical College and Institute of Acupuncture Research (WHO Collaborating Center for Traditional Medicine), Institute of Brain Science, Fudan University, 200032 Shanghai, China; ^4^ Institute of Integrative Medicine of Fudan University, 200032 Shanghai, China; ^5^ Department of Gynecology, Obstetrics and Gynecology Hospital of Fudan University, 200011 Shanghai, China; ^6^ Shanghai Key Laboratory of Female Reproductive Endocrine Related Diseases, 200011 Shanghai, China

**Keywords:** PCOS, hyperandrogenism, insulin resistance, hepatic metabolism, inflammation

## Abstract

Women with polycystic ovary syndrome (PCOS) are at high risk for nonalcoholic fatty liver disease (NAFLD). While insulin resistance is a common trait for both PCOS and NAFLD, hyperandrogenism is also considered to be a key factor contributing to PCOS, and the molecular mechanisms behind the interactions between insulin resistance and hyperandrogenism in the female liver remain largely unexplored. Using chronic treatment with insulin and/or human chorionic gonadotropin (hCG), we showed that all female rats with different treatments induced imbalance between *de novo* lipogenesis and mitochondrial β-oxidation via the *Pparα/β–Srebp1/2–Acc1* axis, resulting in varying degrees of hepatic steatosis. Given the fact that hepatic lipid metabolism and inflammation are tightly linked processes, we found that hCG-induced hyperandrogenic rats had strongly aggravated hepatic inflammation. Further mechanistic investigations revealed that dysregulation of the IRS–PI3K–Akt signaling axis that integrated aberrant inflammatory, apoptotic and autophagic responses in the liver was strongly associated with hyperandrogenism itself or combined with insulin resistance. Additionally, we found that hCG-treated and insulin+hCG-induced rats developed visceral adipose tissue inflammation characterized by the presence of “crown like” structure and increased inflammatory gene expression. Because a more pronounced hepatic steatosis, inflammatory responses, and hepatocyte cell damage were observed in insulin+hCG-induced PCOS-like rats, our finding suggest that NAFLD seen in PCOS patients is dependent of hyperandrogenism and insulin resistance.

## INTRODUCTION

Nonalcoholic fatty liver disease (NAFLD) is an asymptomatic chronic liver disease, and it is thought to affect 25%–45% of the adult population worldwide and is recognized as the leading cause of liver dysfunction in Western societies [1]. NAFLD is typically characterized by more than 5.5% accumulation of hepatocellular lipids, which sensitizes the liver to inflammation and injury [2]. NAFLD-induced hepatocellular injury presents a wide clinical spectrum ranging from simple steatosis to nonalcoholic steatohepatitis and cirrhosis [2–3]. Evidence for a link between NAFLD and female reproduction has emerged from a population-based study showing that otherwise healthy pregnant women with NAFLD have an increased risk for preeclampsia and gestational diabetes [3]. Recent studies indicate that women with polycystic ovary syndrome (PCOS) have an increased prevalence of NAFLD [4–5]. One important goal of research is to identify novel and specific biomarkers, which can be used for accurate prediction of and treatment with NAFLD in PCOS patients. However, this has been hampered by a lack of understanding of the mechanisms underlying the development of hepatic steatosis and aberrant inflammatory responses in the female liver.

PCOS is a heterogeneous hormone-imbalance gynecological disorder with an estimated prevalence of 4%–21% among adolescent and reproductive-aged women depending on the criteria used to define PCOS and on the ethnicity of the group being studied [6]. PCOS has a multifaceted etiology and pathophysiology and involves both endocrine and reproductive aberrations such as hyperandrogenism and metabolic disturbances such as insulin resistance, hyperinsulinemia, and hyperlipidemia [7]. The relationship between NAFLD and female reproductive diseases has received much attention in medical research recently. While the molecular mechanisms of androgen-dependent suppression of lipid accumulation, cholesterol synthesis, and hepatic cell injury in males are gradually being well characterized and understood [8], various animal studies indicate that the effects of androgens on the inhibition of NAFLD development and progression are inconsistent [8]. Although some evidence, such as the combined effects of hyperandrogenism and diet-induced obesity on hepatic steatosis and liver cell damage in female rodents [9–10], the effects of hyperandrogenism itself on lipid accumulation and inflammation in the female liver are unknown [11]. Because insulin resistance is likely to be an integral pathogenic mechanism of both PCOS and NAFLD [6, 12–13], it is necessary to determine whether hyperandrogenism acts independently or additively with insulin resistance to cause the hepatic steatosis and eventual steatohepatitis of the liver that occurs in women with PCOS.

Rodent models treated with insulin and/or human chorionic gonadotropin (hCG) offer the potential to study the cellular and molecular processes in response to hyperinsulinemia, and hyperandrogenism alone or in combination [14–16]. Additional animal studies from our laboratory and others have shown that rodents co-treated chronically with insulin and hCG exhibit endocrine, metabolic, and reproductive alterations that mimic those in PCOS in humans [14–19]. To our knowledge, a direct comparison of the effects of hyperinsulinemia and/or hyperandrogenism in the female liver has never been carried out. Therefore, the aims of the present study was to determine which of the conditions of hyperinsulinemia and/or hyperandrogenism induce hepatic steatosis and inflammation *in vivo* and to what extent, if any, the resulting phenotype leads to the dysregulation of lipid metabolism, impairment in insulin-mediated IRS–PI3K–Akt signaling, and changes to inflammatory, apoptotic and autophagic responses as well as hepatocyte cell injury.

## RESULTS

### Analysis of metabolic and endocrine abnormalities in rats treated with insulin and/or hCG

**Figure 1:**
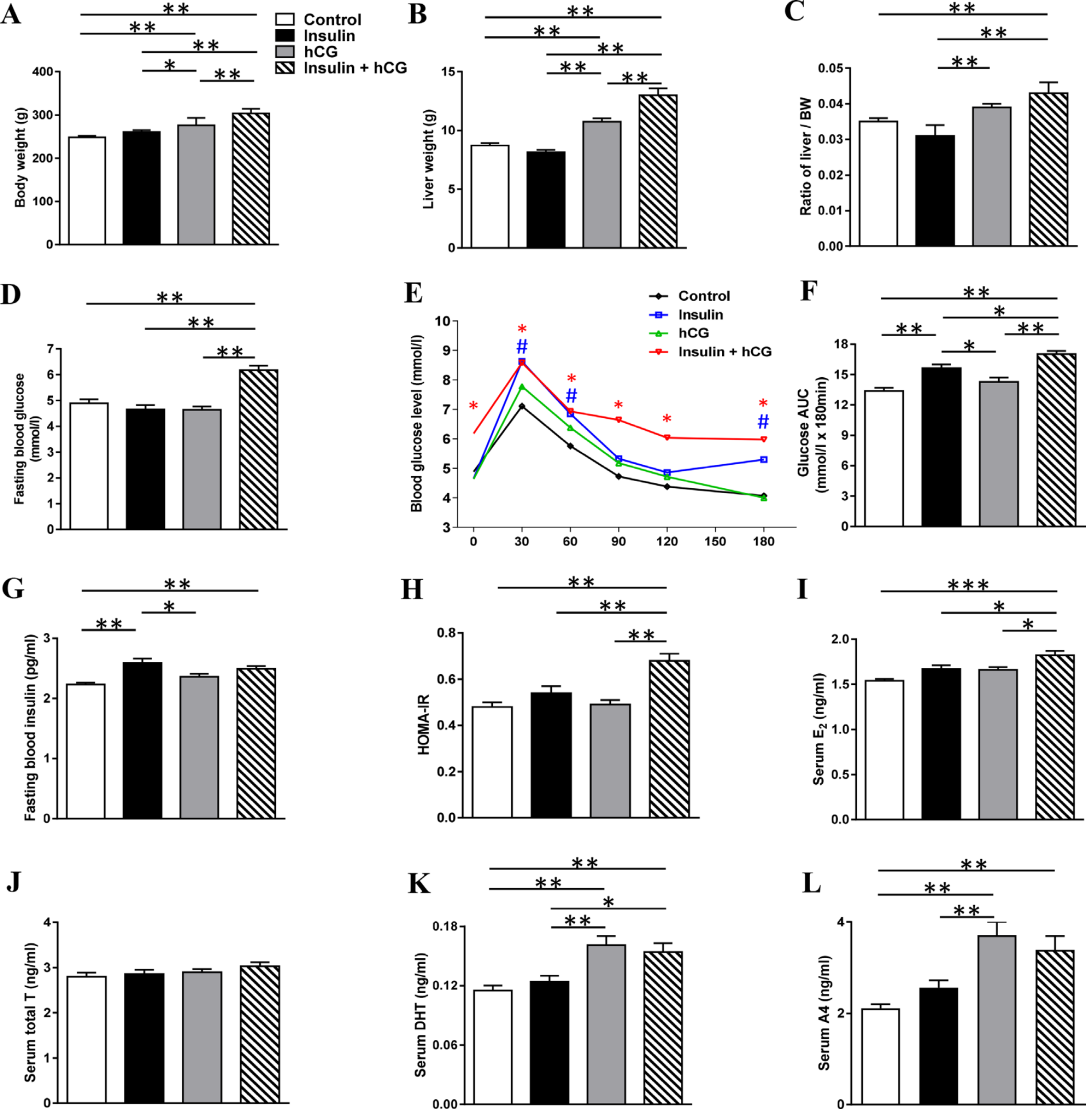
Alterations of metabolic and endocrine parameters in rats treated with insulin and/or hCG Comparison of body weight (**A**), liver weight (**B**), ratio of liver weight to body weight (**C**), fasting blood glucose (**D**), OGTT (**E**), area under the curve (AUC) for glucose (**F**), fasting blood insulin (**G**), HOMA-IR (**H**), serum E_2_ (**I**), T (**J**), DHT (**K**), and A4 (**L**). AUC was calculated by the formula [0.5 × (BG0 + BG30)/2 + 0.5 × (BG30 + BG60)/2 + 0.5 × (BG60 + BG120)/2 + 0.5 × (BG120 + BG180)/2] where the BG terms are the blood glucose levels at 0 min, 30 min, etc. Data in A–L represent the means of *n* = 12/group ± SEM. ^*^p < 0.05; ^**^p < 0.01; ^***^p < 0.001. In the OGTT, ^#^*p* < 0.05 (the insulin group versus the control group) and ^*^p < 0.05 (the insulin+hCG group versus the control group) were determined by analysis of variance (ANOVA) comparing controls to the different treatments. In the HOMA-IR, ^**^p < 0.01 versus the insulin+hCG group.

To establish the experimental model of hyperinsulinemia, hyperandrogenism, and hyperandrogenism with peripheral insulin resistance *in vivo*, we chronically treated rats with insulin and/or hCG. While there was no significant difference in body mass or liver weight between the saline-treated and insulin-treated groups, both the hCG-treated and insulin+hCG-treated rats showed increased body mass and increased liver weight (Figure 1A–1C). As shown in Figure 1, a significant increase in fasting glucose levels was only observed in the insulin+hCG-treated rats (Figure 1D). During the OGTT, there was a significant increase in glucose levels in the insulin-treated rats compared to the control rats after 30 min, 60 min, and 180 min (Figure 1E). Of note, the insulin+hCG-treated rats had reduced glucose tolerance compared to control rats, and this was also shown with the area under the curve measurement (Figure 1E and 1F). A rise in glucose levels at 30 min was observed in insulin-treated and insulin+hCG-treated rats, indicating the hepatic glucose production is shut down by insulin. In contrast to the hCG-treated group, both the insulin-treated and insulin+hCG-treated rats showed significant increases in fasting insulin levels (Figure 1G). However, only insulin+hCG-treated rats had a higher HOMA-IR value than the other three experimental groups (Figure 1H).

The insulin+hCG-treated rats had high E2 levels compared to the other three groups (Figure 1I). While there was no significant difference in circulating T levels between any of the groups (Figure 1J), the hCG-treated and insulin+hCG-treated rats had increased circulating DHT and A4 levels compared to control rats (Figure 1K and 1L), showing the presence of hyperandrogenism in these rats. Western blot analysis revealed that the expression of liver androgen receptor was decreased in insulin-treated and hCG-treated rats compared to insulin+hCG-treated rats (Supplementary Figure 1). Taken together, these data indicate that the insulin+hCG-treated rats display not only hyperandrogenism but also peripheral insulin resistance compared to hCG-treated hyperandrogenic rats.

### Rats treated with insulin and hCG alone or in combination develop hepatic steatosis

**Figure 2:**
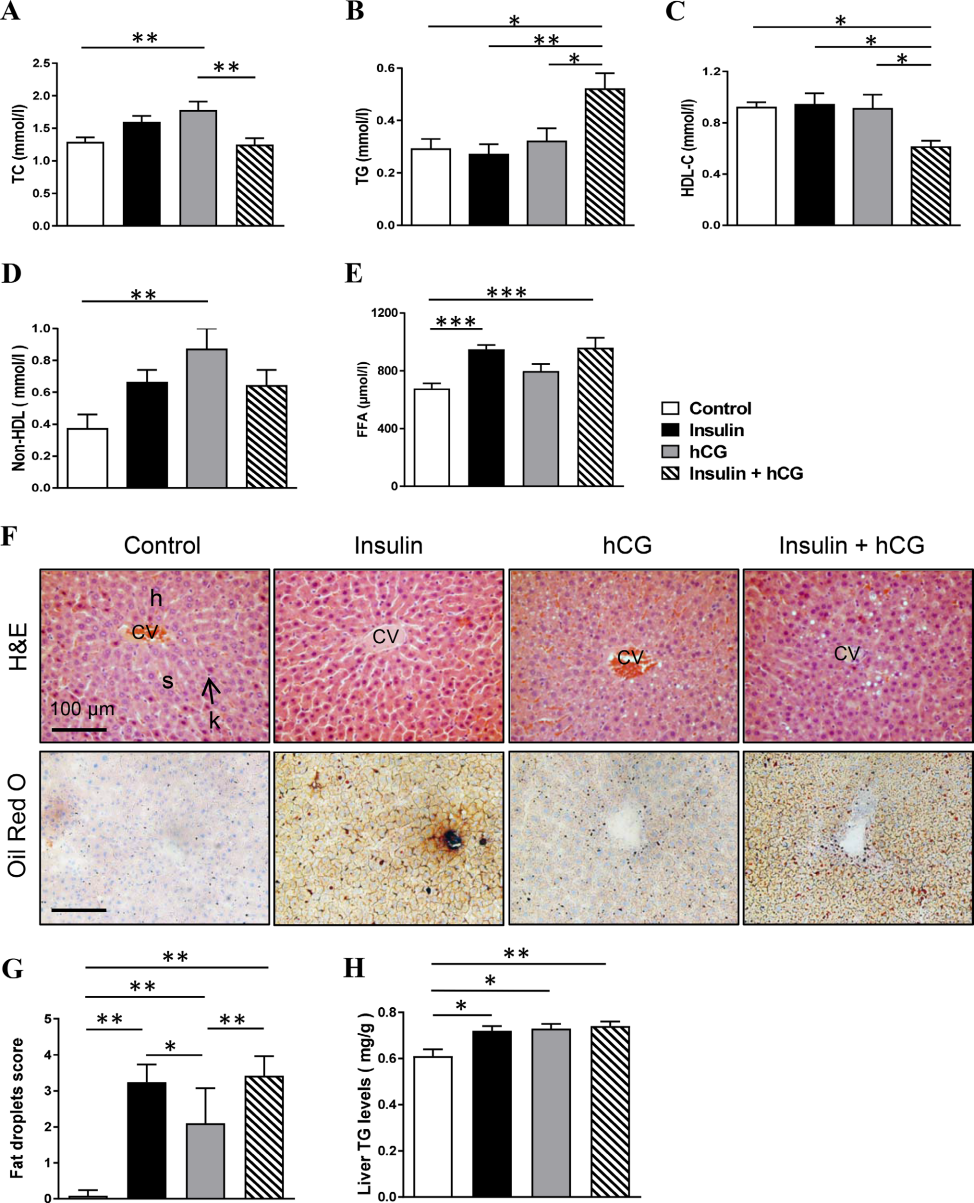
Effects of insulin and/or hCG on major markers of lipid profile, and liver morphology Comparison of circulating TC (**A**), TG (**B**), HDL-C (**C**), non-HDL (**D**), FFA (**E**), and liver TG (**H**). Data represent the means of *n* = 12/group ± SEM. ^*^p < 0.05; ^**^p < 0.01; ^***^p < 0.001. Representative photomicrographs are of liver tissue sections stained with hematoxylin and eosin (H&E) staining (**F**, upper panels) and Oil Red O staining (F, lower panels) with quantification (**G**). Control rats show a normal arrangement of hepatocytes (H) and sinusoids (s) as well as normal central canal and phagocytic Kupffer cells (k). The investigators were blinded to the allocation for morphological analyses (*n* = 6–9/group). Scale bars are indicated in the photomicrographs. CV, central vein.

**Figure 3:**
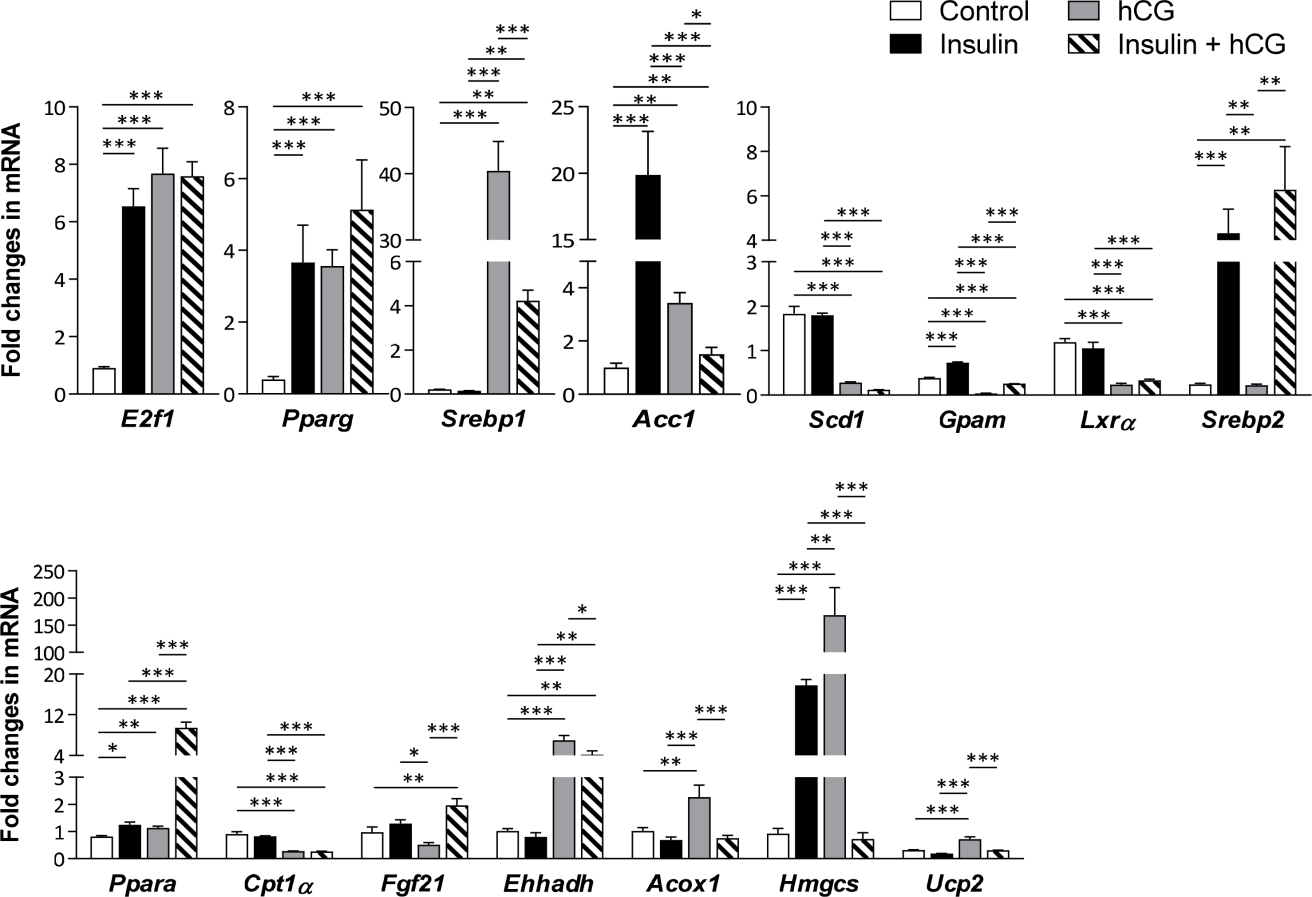
Effects of insulin and/or hCG on the expression of genes involved in hepatic lipid metabolism Relative levels of key regulator mRNAs of hepatic lipid metabolism were determined by qRT-PCR. Data represent means of *n* = 5–6/group ± SEM. ^*^p < 0.05; ^**^p < 0.01; ^***^p < 0.001.

Next, we sought to determine whether rats with hyperandrogenism and/or peripheral insulin resistance develop hepatic steatosis *in vivo*. As shown in Figure 2, hCG-treated rats had increased circulating TC and non-HDL levels, and insulin+hCG-treated rats had increased circulating TG levels in parallel with decreased circulating HDL-C levels (Figure 2A–2D). Further, circulating FFA levels were increased in insulin-treated and insulin+hCG-treated rats compared to the control group (Figure 2E). We performed Oil Red O staining to visualize the morphology and distribution of hepatic lipids in the liver tissue (Figure 2F). There was a significant increase in lipid droplets in hCG-treated rats but an even greater increase in lipid droplets in insulin-treated rats (Figure 2G) consistent with the biochemical measurement of liver TG (Figure 2H). Furthermore, we found that the expression of *E2f1* mRNA was increased in all treated rats compared to control rats (Figure 3).

The increased fat accumulation in rats treated with insulin and/or hCG promoted us to determine whether genes that are involved in the de novo lipogenesis, hepatic TG synthesis, fatty acid β-oxidation, and cholesterol metabolism were altered in rat livers. As shown in Figure 3, although the liver *Pparg*, and *Acc1* mRNA levels were increased regardless of treatment with insulin and/or hCG, *Srebp1* mRNA level was only increased in hCG-treated and insulin+hCG-treated rats. While the up-regulation of *Ppara* mRNA expression was found in all treated rats compared to control rats, a considerable heterogeneity in the *Ppara*-targeted gene expression patterns [20] was observed in the same treatment group. For instance, except *Hmgcs* mRNA level, there was no significant difference in *Cpt1α*, *Fgf21*, *Ehhadlh*, and *Acox1* mRNA levels between insulin-treated and control rats. While *Cpt1α* mRNA level were decreased, we found that *Ehhadh*, *Acox1*, and *Hmgcs* mRNAs were increased in the hCG-treated rats, and *Fgf21* and *Ehhadh* mRNAs were increased in the insulin+hCG-treated rats. Furthermore, the up-regulation of *Srebp2* mRNA expression was only found in insulin-treated and insulin+hCG-treated rats.

### Molecular mechanisms of the regulation of hepatic steatosis in rats treated with insulin and/or hCG

**Figure 4:**
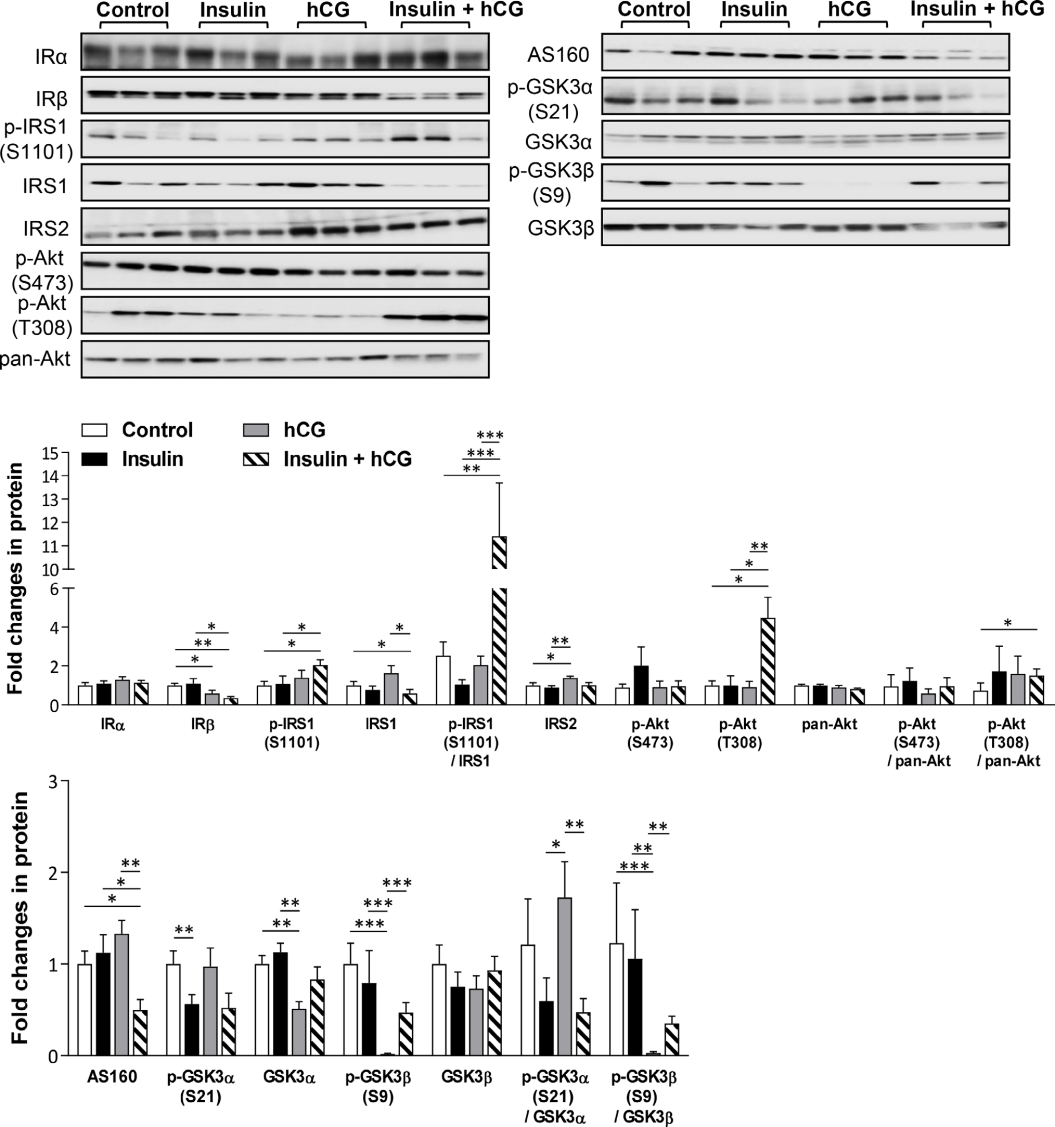
Effects of insulin and/or hCG on the insulin-mediated IRS–PI3K–Akt signaling pathway in the rat liver Representative images with immunoblotting quantification (*n* = 9/group) are shown. Total proteins served as the loading control. Values are expressed as means ± SEM. ^*^p < 0.05; ^**^p < 0.01; ^***^p < 0.001.

Impaired phosphorylation of the insulin receptor and aberrant phosphorylation of IRS1/2 and downstream targets (e.g. Akt) of the insulin signaling cascade results in an abnormal lipid and glucose metabolism in the liver [21–22]. As shown in Figure 4, hCG-treated rats displayed increased IRS2 and decreased IRβ and p110-PI3K (data not shown) protein levels. Previous study reported that in contrast to tyrosine phosphorylation, serine phosphorylation of IRS1 negatively regulates IRS1 function and inactivates insulin-mediated PI3K–Akt signaling pathway [23]. We found that the level of p-IRS1 (S1101) and the ratio of p-IRS1 (S1101):IRS1 were increased, whereas IRβ, and IRS1 protein levels were decreased in insulin+hCG-treated rats compared to control rats. Furthermore, there is evidence that the phosphorylation of GSK3α and GSK3β leads to deactivation of GSK3 and inhibition of glycogen synthase and subsequently results in the suppression of glycogen synthesis in the liver [24]. We showed that the levels of GSK3 (GSK3α and p-GSK3β, an inactivated form of GSK3 β) and the ratio of p-GSK3β:GSK3β were only decreased in hCG-treated rats compared to control rats (Figure 4), indicating that most GSK3β is constitutively active for glycogen synthesis in hCG-treated rats. Although the level of p-Akt (T308) in parallel with the ratio of p-Akt (T308):pan-Akt were increased in insulin+hCG-treated rats compared to control rats (Figure 4), and the increased Akt phosphorylation deactivates GSK3α and GSK3β [24], we found that the levels of GSK3α and GSK3β were not significant altered in insulin+hCG-treated rats. However, while AS160 – which is another downstream target of Akt activation – contributes to glucose transporter-mediated glucose uptake, we observed that AS160 protein expression was only decreased in insulin+hCG-treated rats (Figure 4), which reflects increased circulating glucose levels in these animals (Figure 1). These results indicate that overall insulin resistance and impaired hepatic IRS–PI3K–Akt signaling do exist in insulin+hCG-treated rats.

Insulin and IGF-1 receptors can form functional hybrids in response to the metabolic activities of insulin and IGF-1 [25], and we found that neither IGF1R nor IGFBP1 protein expression were different for the different treatments, whereas IGF2R protein expression was increased in the rat liver for all three treatments compared to control rats (Supplementary Figure 2). This indicates that IGF-1 signaling does not compensate for the loss of insulin receptor.

### Rats treated with hCG in the liver exhibit strongly exacerbated inflammation

**Figure 5:**
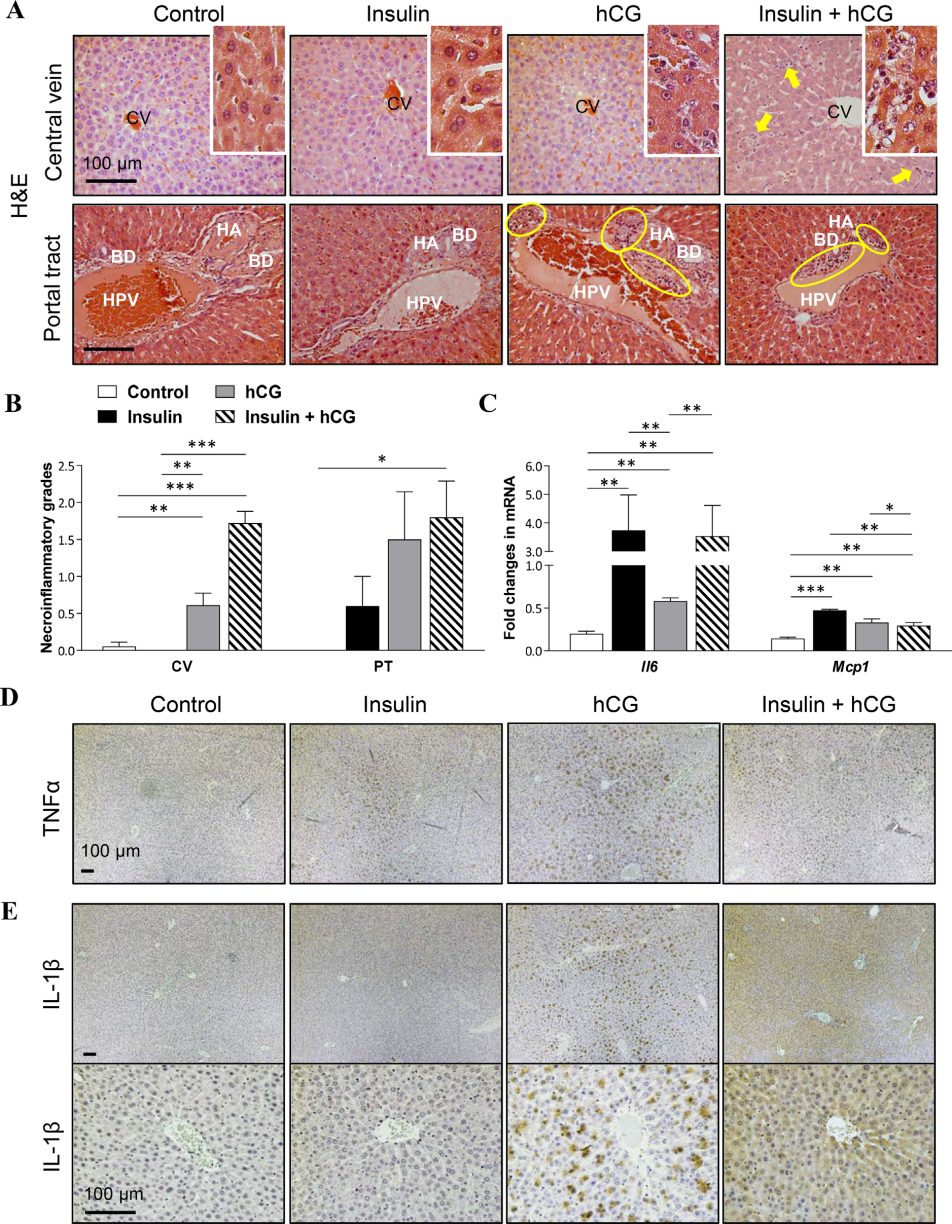
Effects of insulin and/or hCG on hepatic inflammation in the rat liver Representative photomicrographs are of liver tissue sections stained with H&E staining (**A**) with quantification (**B**). Solid bold arrows and rings (yellow) indicate hepatocytes with inflammatory cells. Enhanced magnifications are shown at the right corner of A. The investigators were blinded to the allocation for morphological analyses (*n* = 6–9/group). CV, central vein; PT, portal tract; HA, hepatic artery; HPV, hepatic portal vein; BD, bile duct. Scale bars are indicated in the photomicrographs. Relative levels of mRNAs (**C**) of hepatic inflammatory factors were determined by qRT-PCR. Data in C represent means of *n* = 6/group ± SEM. ^*^p < 0.05; ^**^p < 0.01; ^***^p < 0.001. Immunohistochemical detection of TNFα (**D**) and IL-1β (**E**) in rats treated with insulin and/or hCG. Representative images are shown. The investigators were blinded to the allocation for immunohistochemical analysis (*n* = 5 or 6/group). Scale bars are indicated in the photomicrographs.

Regarding that hepatic steatosis is a low-grade inflammatory response, we set out to study key representative genes and proteins that are involved in the progression of hepatic inflammation [26]. Histological analysis of the liver tissues showed that inflammatory cells were present in hCG-treated and insulin+hCG-treated rats, but not in insulin-treated rats (Figure 5A). In the centrilobular (Figure 5A, upper panels) and periportal (Figure 5A, lower panels) areas of the liver, there was an inflammatory cell accumulation (Figure 5B). Although there were no significant changes in F4/80, MPO, or CD4 protein levels between the different treatment groups (Supplementary Figure 3), we found that a significant increase in *Il-6* and *Mcp1* mRNAs and TNFα protein was detected in insulin-treated, hCG-treated, and insulin+hCG-treated rats (Figure 5C), and IL-1β protein expression was more intense in hCG-treated rats than insulin-treated and insulin+hCG-treated rats (Figure 5C). These data indicate that hyperandrogenism is likely required for and contributes to the exacerbation of abnormal hepatic inflammatory responses in female rats.

**Figure 6:**
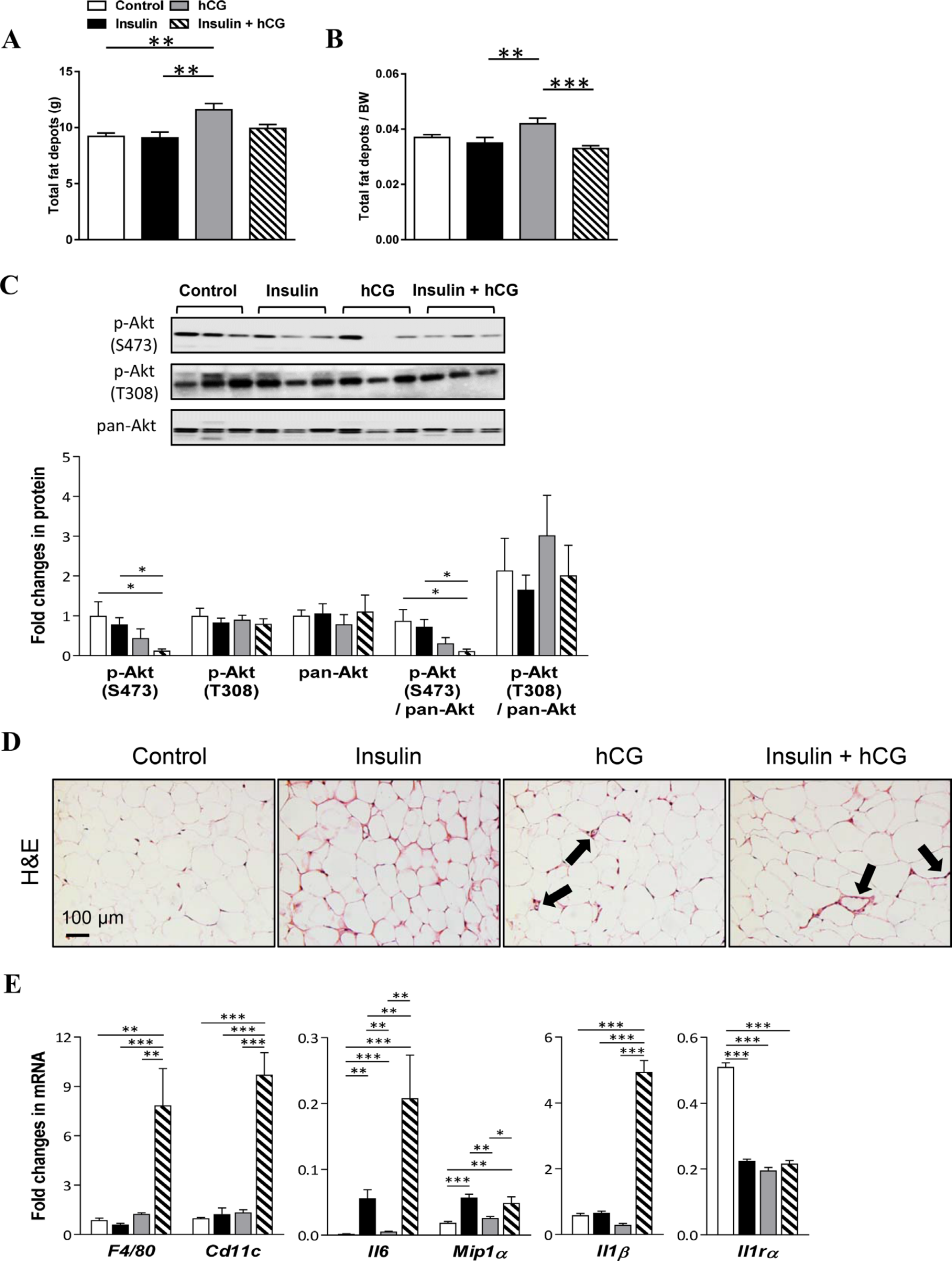
Effects of insulin and/or hCG on adipose tissue distribution, morphological changes, and gene and protein expression in the rat adipose tissue Comparison of total fat depots (**A**), and the ratio of total fat depots to body weight (**B**). Total fat depots include inguinal, parametrical, retroperitoneal, and mesenteric fat portions. Data in A–B are means of *n* = 12/group ± SEM. Relative protein (**C**) and mRNA (**E**) levels of the phosphorylation of Akt and activated inflammatory factors were determined by Western blot analysis and qRT-PCR. For immunoblotting quantification, total proteins served as the loading control. Data in C represent means of *n* = 9/group ± SEM, and data in E are means of *n* = 6/group ± SEM. ^*^p < 0.05; ^**^p < 0.01; ^***^p < 0.001. H&E staining of mesenteric fat tissue sections (**D**) indicates that “crown like” structures are present in rats treated with insulin and hCG (solid bold arrows). Scale bars are indicated in the photomicrographs.

Changes in adiposity can influence the flux of fatty acids to the liver and therefore the degree of liver steatosis [27]. As shown in Figure 6, total fat depots and the ratio of total fat depots to body weight were higher in hCG-treated rats compared to the other experimental groups (Figure 6A and 6B). We showed that treatment with either insulin or hCG did not alter p-Akt (S473 and T308) and pan-Akt protein expression. However, concomitant treatment with insulin and hCG decreased p-Akt (S473) protein expression and p-Akt (S473):pan-Akt compared to the control group (Figure 6C). These results indicate that in insulin+hCG-treated rats, the insulin resistant adipose tissue failed to mount insulin signals to suppress lipolysis, resulting in increased circulating FFA (Figure 2E), possibly flooding the liver with fatty acids and increasing fat accumulation (Figure 2F and 2G). We further evaluated the morphology of the VAT and observed “crown like” structures in the insulin+hCG-treated rats (Figure 6D). We also studied gene and protein expression in the VAT and found that *F4/80* and *Cd11c* mRNAs were increased in insulin+hCG-treated rats (Figure 6E). Considering that adipose tissue served as an essential endocrine and immune organ is capable of producing and secreting multiple cytokines and chemokines [28–29], the expression of genes that are used as markers for the identification of proinflammatory M1-type macrophages (*Il6*, *Mip1α*, and *Il1β*) and anti-inflammatory M2-type macrophages (*Il1rα*) was measured (Figure 6E). We found that the expression of *Il6*, *Mip1α*, and *Il1β* mRNAs was increased in insulin+hCG-treated rats compared to control rats. We also found that the expression of *Il1rα* mRNA was increased in the rat VAT for all three treatments compared to control rats (Figure 6E).

### Suppression of hepatic apoptosis and activation of autophagy in rats treated with insulin and/or hCG

**Figure 7:**
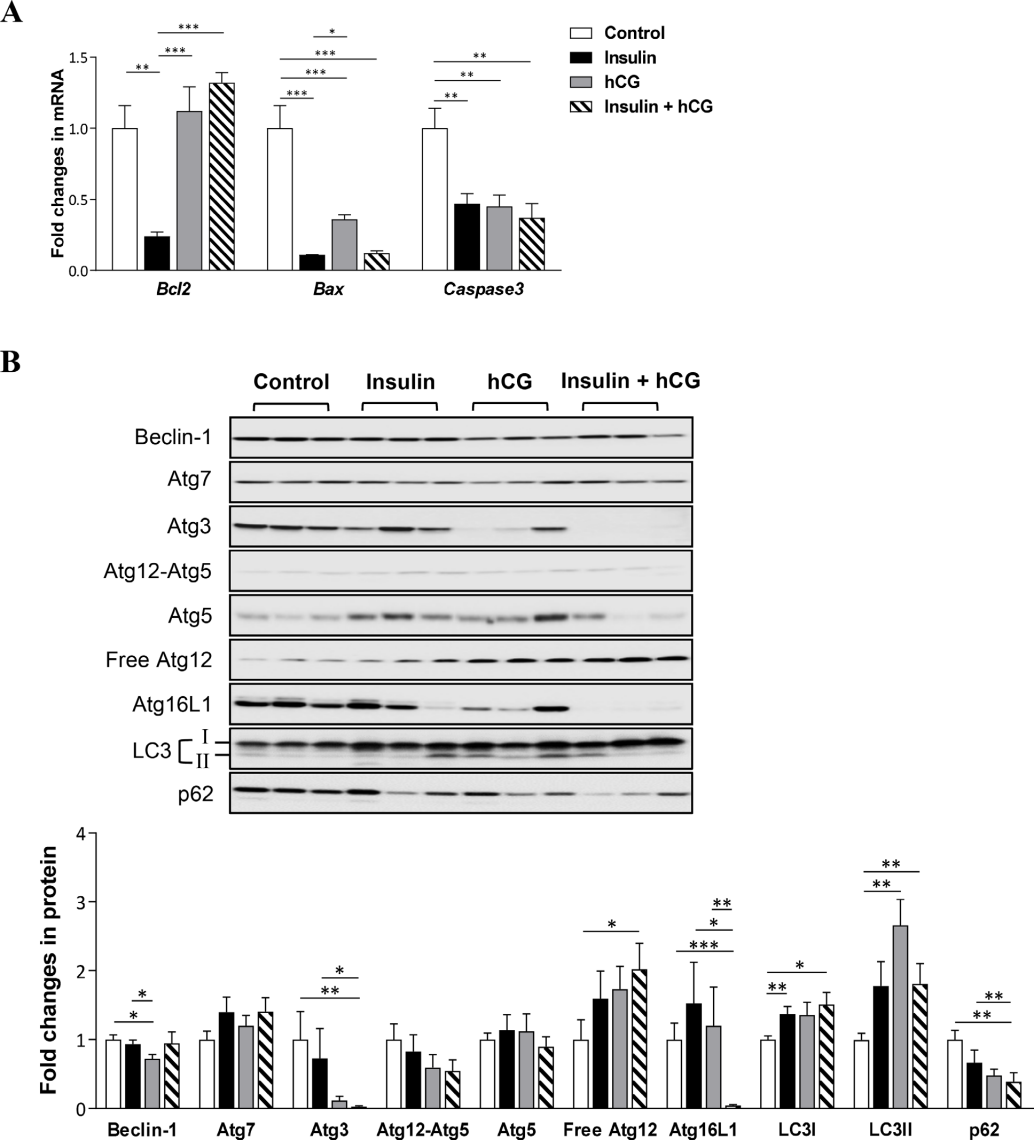
Effects of insulin and/or hCG on apoptosis and autophagy in the rat liver Relative levels of mRNAs (**A**) and proteins (**B**) of pro-apoptotic and anti-apoptotic genes and autophagy-related proteins were determined by qRT-PCR and Western blot analysis. For immunoblotting quantification, total proteins served as the loading control. Data in A represent means of *n* = 6/group ± SEM, and the data in B are means of *n* = 9/group ± SEM. ^*^p < 0.05; ^**^p < 0.01; ^***^p < 0.001.

We used qRT-PCR and Western blot analysis to determine the expression of genes and proteins that are involved in the apoptosis and autophagy processes in rat livers. As shown in Figure 7, *Bcl2*, *Bax*, and *Caspase3* mRNA expression was decreased in insulin-treated rats compared to controls. While there was no significant alteration of *Bcl2* mRNA expression, decreased *Bax and Caspase3* mRNA expression was detected in hCG-treated and insulin+hCG-treated rats (Figure 7A). Next, we found that the expression of major components of the autophagy process, including Beclin-1, Atg7, Atg3, Atg12-Atg5, free Atg12, Atg16L1, and LC3II was not affected by hyperinsulinemia. Although the levels of Beclin-1, Atg3, and Atg16L1 proteins were decreased in hCG-treated and/or insulin+hCG-treated rats compared to control rats (Figure 7B), we observed that increased total cellular LC3, in particular LC3II (an autophagic activity marker), was accompanied by a reduction of level of p62/sequestosome-1 (an autophagic flux marker) [30] in the liver. Of note, increased expression of LC3I and LC3II and decreased expression of p62 were found in insulin+hCG-treated rat livers (Figure 7B). These results demonstrated that hepatic apoptosis and autophagy are oppositely regulated by the presence of hyperandrogenism and insulin resistance.

### Alteration of fibrogenesis-related gene and protein expression in parallel with liver functional impairment, but not induction of fibrosis in rats treated with insulin and/or hCG

**Figure 8:**
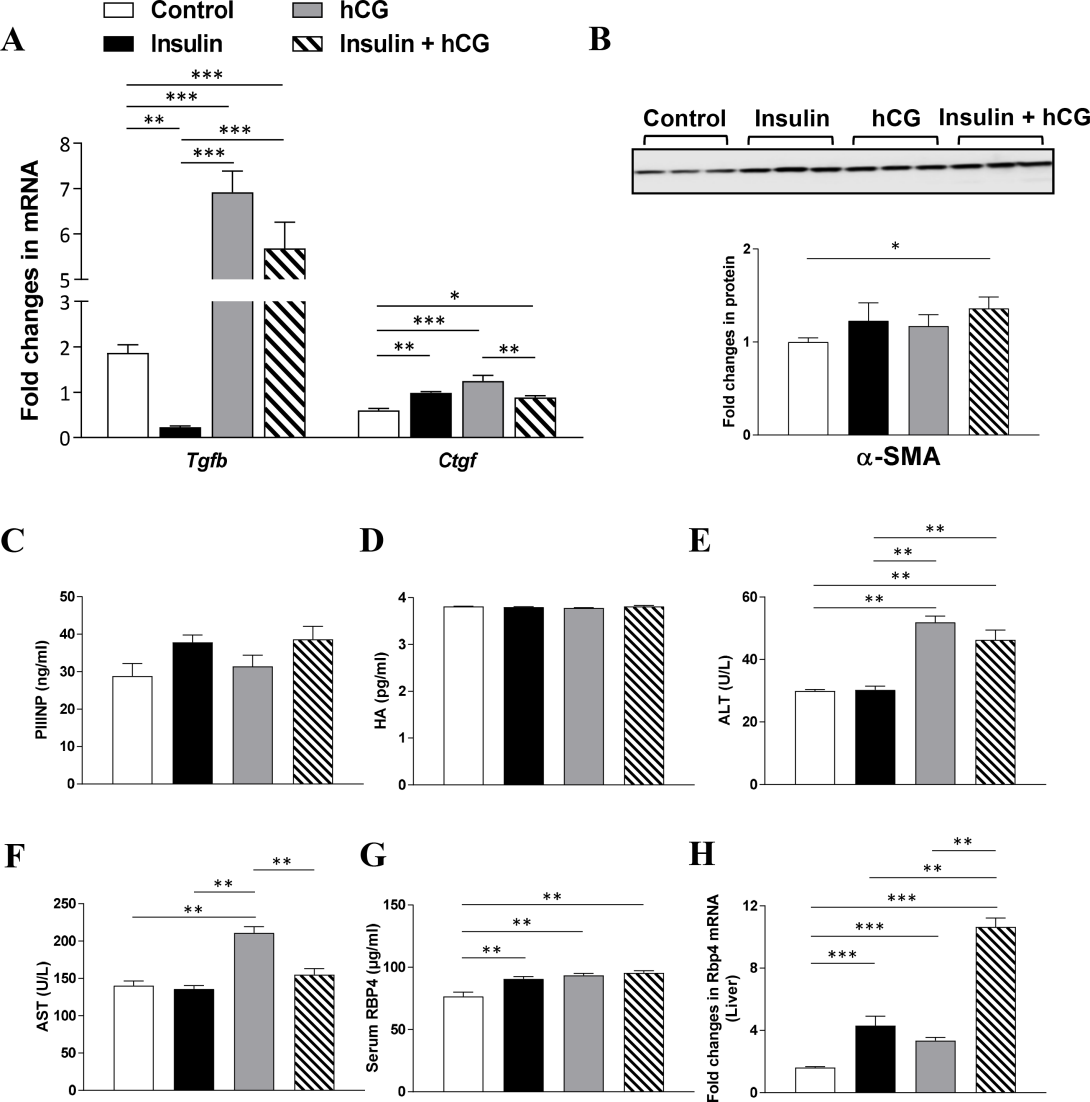
Effects of insulin and/or hCG on fibrosis-related gene and protein expression, and liver functional markers in the rat liver Relative levels of mRNAs (**A**) and protein (**B**) of major markers for hepatic stellate cell activation were determined by qRT-PCR and Western blot analysis. For immunoblotting quantification, total proteins served as the loading control. Data in A represent means of *n* = 6/group ± SEM, and data in B are means of *n* = 9/group ± SEM. Comparison of circulating PIIINP (**C**), HA (**D**), ALT (**E**), AST (**F**), RBP4 (**G**), and liver RBP4 (**H**) are shown. Data in C–H represent means of *n* = 12/group. ^*^p < 0.05; ^**^p < 0.01; ^***^p < 0.001.

Finally, we assessed the induction of liver fibrosis by treatment with insulin and/or hCG. As shown in Figure 8, insulin-treated rats had significantly decreased *Tgfb* mRNA expression compared to controls. In contrast, both hCG-treated and insulin+hCG-treated rats had increased *Tgfb* mRNA expression compared to the saline-treated and insulin-treated rats (Figure 8A). We observed that chronic treatment with insulin and/or hCG significantly increased *Ctgf* mRNA levels in the liver (Figure 8A). Furthermore, increased α-SMA protein expression was only observed in insulin+hCG-treated rats (Figure 8B). We completed our study by examining whether alterations in collagen synthesis were associated with the induction of liver fibrosis. The analysis of circulating PIIINP and HA levels (Figure 8C and 8D) and liver hydroxyproline levels (Supplementary Figure 4A) together with picrosirius red and Masson's trichrome staining on live tissues (Supplementary Figure 4B and 4C) revealed no evidence of significant induction of fibrosis in any of the groups. However, increased circulating levels of ALT and/or AST – which are indicators of hepatocellular damage [31] – were observed in hCG-treated and insulin+hCG-treated rats compared to saline-treated and insulin-treated rats (Figure 8E and 8F), and this indicated that hCG treatment but not insulin treatment resulted in signs of hepatocyte cell injury.

RBP4 is mainly synthesized in hepatocytes followed by other tissues/cells, including adipose tissue, under normal conditions [32]. Several clinical studies have shown that increased circulating levels of RBP4, which mainly functions as a specific transport protein of retinol (vitamin A) in the circulation, are associated with either hyperandrogenism [33–35] or insulin resistance [36–37] in women with PCOS. We therefore tested the effect of hyperinsulinemia, hyperandrogenism, or the combination of hyperandrogenism and insulin resistance on RBP4 expression. We showed that chronic treatment with insulin and/or hCG significantly increased RBP4 levels in the circulation (Figure 8G) and liver (Figure 8H).

## DISCUSSION

Elucidating the PCOS-related metabolic and endocrine conditions causing hepatic steatosis, aberrant inflammatory responses, and hepatic cell injury have been considered as a key for understanding the cellular and molecular mechanisms behind the development of NAFLD in PCOS patients. Here we report for the first time that hyperinsulinemia, hyperandrogenism, and a combination of hyperandrogenism and insulin resistance all contribute to development of hepatic steatosis as a result of impairment of lipid metabolism in the female rat liver. We show that dysregulation of insulin-mediated IRS–PI3K–Akt signaling pathway results in an imbalance between *de novo* lipogenesis and mitochondrial β-oxidation, as well as impaired hepatic gluconeogenic pathway, thereby exacerbating fat accumulation and the resultant hepatic steatosis. Furthermore, we have demonstrated that hyperandrogenism itself or combined with insulin resistance facilitate inflammatory response, and hepatocyte cell damage, which is involved in the regulation of apoptotic and autophagic processes, rather than induction of liver fibrosis in female rats.

Because it is difficult to provide any direct experimental proof or molecular mechanistic evidence that the insulin resistance and/or hyperandrogenism in women with PCOS is responsible for the onset and progression of NAFLD, animal models might provide insight into the separate role of hyperinsulinemia and hyperandrogenism in the development of hepatic steatosis and aberrant inflammatory responses in the female liver. It is well known that both insulin resistance and hyperandrogenism are the key clinical feature of PCOS [7]. Having determined that insulin-treated or hCG-treated rats do not develop a combination of insulin resistance and hyperandrogenism, insulin+hCG-treated rats were used to examine whether aberrant lipid metabolism and hepatic dysfunction occurred when both conditions were present in a similar manner as in PCOS patients.

One of the novel findings in this paper is that insulin+hCG-treated rats exhibit the hallmarks of metabolic syndrome and hepatic cell damage, including increased body and liver weight, increased circulating FFA, TG, fasting insulin, and ALT levels, and decreased circulating HDL-C levels, all of which are consistent with the metabolic and liver dysfunction pattern seen in hyperandrogenic PCOS patients with NAFLD [38–40] and in DHT-induced PCOS-like rodents [9, 41]. E2F1, a transcription factor, has been shown to be critical for the inhibition of hepatic fat accumulation *in vivo* [42]. Although the liver TG and *E2f1* mRNA levels are increased regardless of treatment with insulin and/or hCG, our morphological analysis indicates that hepatic lipid accumulation is greater in insulin-treated and insulin+hCG-treated rats than hCG-treated rats. It is well-known that hepatic steatosis reflects an imbalance between lipoprotein and fatty acid uptake, fatty acid synthesis and oxidation, and lipoprotein secretion [27]. The use of qRT-PCR analysis demonstrates that the expression patterns of genes that are important for hepatic lipid metabolism are not the same between the different experimental groups. Interestingly, hepatic *Srebp1* mRNA expression is increased in ovariectomized mice treated with DHT [9], and we found similar upregulation in hCG-treated and insulin+hCG-treated rats, which show the increased endogenous androgen levels. We also found that hepatic *Srebp2* mRNA expression was increased in insulin-treated and insulin+hCG-treated rats. These findings suggest that the mRNA expression of *Srebp1* and *Srebp2* – which play critical roles in fatty acid and cholesterol synthesis in the liver – can be regulated by androgens or insulin. Tissue/cell-specific PPARα and PPARβ appear to have different but overlapping functions in hepatic lipid metabolism [43–44]. We observed increases in both *Ppara* and *Pparg* mRNA expression in rats regardless of treatment with insulin and/or hCG, and this suggests that the induction of varying degrees of hepatic steatosis by the different treatments depends on the balance between PPARα and PPARβ activity. ACC is one of key enzymes for hepatic *de novo* lipogenesis [45]. Our studies indicate that the increased *Acc1* mRNA levels associated with up-regulation of *Srebp1* and *Pparg* mRNA levels are likely to cause an accumulation of hepatic TG in parallel with down-regulation of *Cpt1α* mRNA level, resulting in suppression of β-oxidation in female rat liver in the presence of hyperandrogenism and insulin resistance. In our measurements of the protein expression associated with the insulin-mediated IRS–PI3K–Akt signaling cascade, we found that the insulin+hCG-treated rats have decreased hepatic IRβ, IRS1, and AS 160 protein expression compared to saline-treated or insulin-treated rats. These data are consistent with liver-specific IR knockout studies showing that loss of insulin signaling in hepatocytes results in severe insulin resistance and progressive hepatic dysfunction [46]. Our data suggest that the impairment in insulin-mediated IRS–PI3K–Akt signaling promotes lipid accumulation and hepatic steatosis, which in turn contribute to chronic hepatic inflammation in the female liver under the conditions of hyperandrogenism and insulin resistance.

In this study, the hCG-treated rats did not show peripheral insulin resistance and marked hyperglycemia. These rats, however, showed the development of hepatic steatosis. Furthermore, liver histological inflammation was associated with the significant alterations of hepatic TNFα and IL-1β protein expression in parallel with the increased levels of circulating TC, ALT and AST, especially in these hyperandrogenic rats. Thus, it is likely that hyperandrogenism plays a more profound role in inflammatory liver injury compared to hyperinsulinemia in females.

Although insulin resistance is linked to pathogenesis of NAFLD [13], reports on the linkage of insulin resistance and NAFLD in PCOS patients have been controversial. Three studies have found that insulin resistance is associated with hepatic steatosis in PCOS patients [4, 47–48], but two other studies have reported that no such association exists in PCOS patients [4, 49]. Disagreements in findings might reflect differences in the underlying characteristics of PCOS patients or problems with not using liver biopsies for defining NAFLD. Because insulin-treated rats exhibit hepatic steatosis and different liver gene and protein expression profiles compared to hCG-treated, insulin+hCG-treated, and control rats, it is tempting to speculate that synergistic action of hyperinsulinemia and hyperandrogenism might be present in PCOS patients with NAFLD.

Another interesting finding in this paper is that hyperandrogenism and insulin resistance significantly regulate apoptosis and autophagy in the female liver. The liver is composed of hepatocytes, Kupfferr cells, hepatic stellate cells (HSCs), endothelial cells, and biliary cells [1], and the cell-to-cell interaction and transition are essential for controlling hepatic inflammation and liver injury through the different mechanisms such as cell proliferation, apoptosis and autophagy [50–52]. We employed qRT-PCR to probe hepatic expression of genes associated with anti-apoptotic (*Bcl2*) and pro-apoptotic (*Bax* and *caspase3*) processes. Our data indicate that hyperandrogenism or combined with insulin resistance likely decreases hepatic apoptosis in female rats. Furthermore, we found a dynamic pattern of autophagic activation in rats under the conditions of hyperandrogenism and insulin resistance. In contrast to the decreased several autophagy gene expression and suppression of autophagy activity in the liver obtained from the mouse model of long-term diet-induced obesity [53], we found that the increased LC3II (an autophagic activity marker) protein levels were associated with down-regulation of p62 protein expression in hCG-treated and insulin+hCG-treated rats compared to control rats. We propose that this difference may be due to the possibility that the hepatic cell injury in rats treated with insulin and/or hCG is likely to be early stage in NAFLD [54]. In fact, obesity-induced insulin resistance increases hepatic autophagy during the early development of NAFLD [55]. Because inflammatory liver injury links defective hepatic autophagy is associated with NAFLD [50–51], and LC3 participates in intracellular lipid droplet formation [56], whether the increased hepatic autophagy initially contributes to fat accumulation in the female rat liver requires further study.

In the normal liver, HSCs remain in a quiescent state and are activated by damaged hepatocytes or other liver cells, and this activation contributes to the development of liver fibrosis [26]. It is interesting to note that the expression of α-SMA, a protein marker for the myofibroblast-like cells, is increased in insulin+hCG-treated rats. However, such upregulation of the α-SMA protein level is not observed in either insulin-treated or hCG-treated rats. This suggests that HSCs undergoing the dynamic shift from the quiescent state to the activated state require the presence of both insulin resistance and hyperandrogenism *in vivo*. Thus, whether the activation of HSCs plays a central role in the regulation of apoptosis and autophagy in the female liver under the conditions of hyperandrogenism and insulin resistance requires further investigation. Although we failed to detect any significant induction of collagen production or fibrosis in these animals, we are aware that high-fat diet and longer time course studies using the same treatment protocol will allow us to determine whether the activation of HSCs by persistent hepatic inflammation results in progressive fibrosis and the loss of hepatic function in female rats.

The limitations of the current study include the PCOS-like rat model and the lack of confirmation of the results using human samples. Despite the fact that insulin+hCG-treated rats have served as a suitable animal model for studying PCOS-related reproductive function, we found that the expression of several genes reported to be PPARα- and PPARγ-dependent was not altered, even was oppositely regulated in insulin+hCG-treated rat liver. We note that combined treatment with insulin and hCG not only induced peripheral insulin resistance and hyperandrogenism, but also increased estrogen levels in female rats (Figure 1). Because estrogen suppresses the increased liver fat accumulation in ovariectomized mice [57], and such inhibitory effect appears to be mediated by regulating the expression of PPARα-targeted genes [58], it is likely that heterogeneity in the gene expression patterns in the insulin+hCG-treated rat liver may represent an extensive crosstalk and some redundancy between androgen/androgen receptor signaling and estrogen/estrogen receptor signaling.

In summary, this study presents several lines of *in vivo* evidence that hyperinsulinemia and hyperandrogenism, either alone or in combination with insulin resistance, alter hepatic lipid metabolism, liver inflammatory responses, and cellular function and that the effects of the different conditions are distinct from each other. By deciphering the metabolic, endocrine, and molecular alterations along with morphological changes, our findings offer a new understanding of how hyperandrogenism itself or combined with insulin resistance contributes to liver damage in women with PCOS.

## MATERIALS AND METHODS

### Reagents and antibodies

hCG was from NV Organon (Oss, Holland). Human recombinant insulin (Humulin NPH) was from Eli Lilly Pharmaceuticals (Giza, Egypt). 3,3-diaminobenzidine tetrahydrochloride (DAB) was from Sigma-Aldrich (St. Louis, MO). The avidin-biotinylated-peroxidase complex detection system was from Vector Laboratories Inc. (Burlingame, CA). The primary antibodies used for Western blot and immunohistochemical analyses in the present study, their dilutions, and their sources are listed in Supplementary Table 1. Anti-mouse IgG horseradish peroxidase (HRP)-conjugated goat (A2304), anti-rabbit IgG HRP-conjugated goat (A0545), and anti-goat IgG HRP-conjugated goat (A5420) secondary antibodies were from Sigma-Aldrich.

### Animal handling, and experimental design

All animals were treated and used according to the National Institute of Health guidelines on the care and use of animals, and the experimental protocols were approved by the Animal Care and Use Committee of the Heilongjiang University of Chinese Medicine, China. Seventy-day-old female Sprague Dawley rats (obtained from the Laboratory Animal Centre of Harbin Medical University, Harbin, China) lived under controlled conditions. Rats were housed individually in a pathogen-free room with controlled temperature (22 ± 2° C) and a 12 h light/dark cycle with free access to fresh drinking water. Animals were maintained on a normal commercial diet containing 23% calories from protein, 10% from fat, and 67% from carbohydrates. The doses and treatment protocols for insulin and hCG are described detail in our previous paper [19]. Briefly, adult rats were randomly assigned to be treated with (A) an equal volume of saline as controls; (B) insulin, which was started at 0.5 IU/day and gradually increased to 6.0 IU/day between the 11th day and the 22nd day to induce hyperinsulinemia and peripheral insulin resistance; (C) 3.0 IU/day hCG to induce hyperandrogenism; or (D) insulin plus hCG to induce a combination of peripheral insulin resistance and hyperandrogenism. Animals were treated twice daily with subcutaneous injections for 22 days. On the 23rd day, the animals were deeply anesthetized and decapitated. Trunk blood was collected and liver and adipose tissues (inguinal, parametrical, retroperitoneal, and mesenteric fats) were dissected and wet weighed. Both tissues and blood samples were collected for histological, molecular, and biochemical analyses.

### Oral glucose tolerance test (OGTT)

The OGTT was performed in live rats on day 22 as described previously [19].

### Histological and immunohistochemical analyses

Liver and adipose tissues were fixed in 10% formalin, paraffin-embedded, and sectioned for hematoxylin and eosin (H&E) staining according to standard procedures. Fibrosis was evaluated by both picrosirius red and Masson's trichrome staining. The presence of lipid droplets was determined by Oil Red O staining in frozen sections that had been counterstained with hematoxylin. Liver lipid accumulation was quantified by scanning five randomly picked fields for each liver section and scoring them as 0 (no fat droplets), 1 (very few fat droplets), 2 (few fat droplets), 3 (multiple fat droplets), and 4 (many fat droplets). Liver inflammation was graded according to the guidelines pathology committee of non-alcoholic steatohepatitis clinic research network [54]. Immunohistochemistry was performed according to previously described methods [59]. The primary antibodies were listed in the Supplementary Table 1. Positive and negative controls were run to validate the picrosirius red and Masson's trichrome staining and all immunohistochemical assays.

### Western blot analysis

Liver and adipose tissues were lysed using RIPA buffer (Sigma-Aldrich) supplemented with cOmplete Mini protease inhibitor cocktail tablets (Roche Diagnostics, Mannheim, Germany) and PhosSTOP phosphatase inhibitor cocktail tablets (Roche Diagnostics). Equal amounts (20 μg) of protein for each treatment group were resolved on 4–15% TGX stain-free gels (Bio-Rad Laboratories GmbH, Munich, Germany) and transferred onto PVDF membranes. The membranes were probed with the primary antibody (Supplementary Table 1) in 0.01 M Tris-buffered saline supplemented with Triton X-100 (TBST) containing 5% nonfat dry milk followed by HRP-conjugated secondary antibody. When necessary, the PVDF membranes were stripped using Restore PLUS Western blot stripping buffer (Thermo Scientific, Rockford, IL) for 15 minutes at room temperature, washed twice in TBST, and then reprobed. Ultraviolet activation of the stain-free gel on a ChemiDoc MP Imaging System (Bio-Rad) was used to control for proper loading [19, 59]. Blots were scanned and quantified using Image Laboratory (Version 5.0, Bio-Rad, Sweden). All specific protein band densities were normalized to the total-protein loading control.

### Quantitative real time-polymerase chain reaction (qRT-PCR) analysis

A detailed explanation of the qRT-PCR protocol has been published elsewhere (Zhang *et al.* 2016). Total RNA was isolated from liver and adipose tissues from each rat using TRIzol reagent (Life Technologies) and reverse transcribed into cDNAs, which were quantified by qRT-PCR using SYBR green qPCR master mix (#K0252, Thermo Scientific, Rockford, IL) and a Roche Light Cycler 480 sequence detection system (Roche Diagnostics Ltd., Rotkreuz, Switzerland) with specific primers listed in Supplementary Table 2. The CT values for both *Gapdh* and *U87* were not significantly different in any of the groups, which confirmed that the loading was similar between the samples. The results for target genes were expressed as the amount relative to the average CT values of *Gapdh* + *U87* in each sample. Relative gene expression was determined with the 2−ΔΔCT method.

### Metabolites and hormones

Serum insulin concentrations (Cloud-Clone Corp., Houston, TX), FFA (non-esterified fatty acids, BioTSZ, Lexington, MA), and serum/tissue RBP4 levels (Abcam, Cambridge, UK) were determined using ELISA kits. Homeostasis model assessment of insulin resistance (HOMA-IR) was calculated to assess changes in insulin sensitivity [60, 61]. Concentrations of glucose, E2, total T, DHT, A4, TC, TG, HDL-C, ALT, AST, PIIINP, and HA were determined enzymatically with a Hitachi 7600 automatic biochemical analyzer (Hitachi Ltd., Osaka, Japan). Intrahepatic TG contents were extracted from liver tissues using a 1:1 (v/v) chloroform/methanol mixture. The prepared sample was then centrifuged at 1200 × *g* for 10 min, and the supernatant was used for lipid measurements. The intra- and inter-assay coefficients of variation are listed in Supplementary Table 3. The intrahepatic collagen content was measured with a hydroxyproline assay kit (Sigma-Aldrich). All assays were performed according to the manufacturer's instructions and with reagents and materials provided by the manufacturer.

### Statistical analysis

Statistical analyses were performed using SPSS version 21.0 for Windows (SPSS Inc., Chicago, IL). Datasets were first assessed for normal distribution using the Shapiro–Wilk test. Differences in outcomes were determined by one-way ANOVA followed by Tukey's post hoc test for normally distributed data or the Kruskal–Wallis test followed by the Mann–Whitney *U*-test for skewed data. Only results with *p* < 0.05 were considered statistically significant. All numeric data are provided as the means ± SEM.

### Genes

*Acc1*, acetyl-CoA carboxylase 1; *Acox1*, acyl-CoA oxidase 1; *Bax*, Bcl2 associated X; *Bcl2,* B-cell lymphoma-2; *Cd11c*, integrin alpha X (Itgax); *Cpt1α*, carnitine palmitoyltransferase 1 alpha; *Ctgf*, connective tissue growth factor; *Gapdh*, glyceraldehyde-3-phosphate dehydrogenase; *Gpam*, mitochondrial glycerol-3-phosphate acyltransferase; *Ehhadh*, enoyl-CoA hydratase and 3-hydroxyacyl CoA dehydrogenase; *E2f1*, E2F transcription factor 1; *Fgf21*, fibroblast growth factor 21; *F4/80*, adhesion G protein-coupled receptor E1 (Adgre1); *Hmgcs*, 3-hydroxy-3-methylglutaryl-CoA reductase; *Il1β*, interleukin-1β; *Il1ra*, interleukin-1 receptor alpha; *Il6*, interleukin 6; *Lxrα*, liver X receptor alpha; *Mcp1*, monocyte chemotactic protein 1 (Ccl2); *Mip1a*, macrophage inflammatory protein 1alpha (Ccl3); *Ppar*, peroxisome proliferator-activated receptor; *Scd1*, stearoyl-coenzyme A (CoA) desaturase-1; *Srebp*, sterol regulatory element-binding protein; *Tgfb*, transforming growth factor beta; *Rbp4*, retinol binding protein 4; *Ucp2*, uncoupling protein 2; *U87*, small nucleolar RNA, C/D box 87 (Snord87).

### Proteins

AR, androgen receptor; Atg, autophagic-related protein; α-SMA, α-smooth muscle actin; Akt, protein kinase B; AS160, the Akt serine/threonine kinase 160; CD4, cluster of differentiation 4; GSK3, glycogen synthase kinase 3; F4/80, adhesion G protein-coupled receptor E1; IGFBP1, IGF binding protein 1; IGF1R, insulin-like growth factor receptor 1; IL1β, interleukin-1β; IR, insulin receptor; IRS, insulin receptor substrate; LC3, microtubule-associated proteins 1A/1B light chain 3; MPO, the heme protein myeloperoxidase; PI3K, phosphoinositide 3-kinase; p62, SQSTM1/sequestosome 1 ; TNFα, tumor necrosis factor α.

## SUPPLEMENTARY MATERIALS FIGURES AND TABLES





## Abbreviations

ALT: alanine aminotransferase
AST: aspartate aminotransferase
A4: androstenedione
DHT: 5α-dihydrotestosterone
ELISA: enzyme-linked immunosorbent assay
E2: 17β-estradiol
FFA: free fatty acid
HA: hyaluronic acid
H&E: hematoxylin and eosin
hCG: human chorionic gonadotropin
HDL-C: high-density lipoprotein cholesterol
HOMA-IR: homeostasis model assessment of insulin resistance
HSCs: hepatic stellate cells
IHC: immunohistochemistry
NAFLD: nonalcoholic fatty liver disease
OGTT: oral glucose tolerance tests
PCOS: polycystic ovary syndrome
PIIINP: procollagen type III amino-terminal peptide
qRT-PCR: quantitative real-time PCR
RBP4: retinol binding protein 4
T: testosterone
TC: total cholesterol
TG: triglyceride
VAT: visceral adipose tissue
WB: Western blot analysis.

## References

[1] Rinella ME (2015). Nonalcoholic fatty liver disease: a systematic review. JAMA.

[2] Hotamisligil GS (2006). Inflammation and metabolic disorders. Nature.

[3] Hagstrom H, Hoijer J, Ludvigsson JF, Bottai M, Ekbom A, Hultcrantz R, Stephansson O, Stokkeland K (2016). Adverse outcomes of pregnancy in women with non-alcoholic fatty liver disease. Liver Int.

[4] Macut D, Tziomalos K, Bozic-Antic I, Bjekic-Macut J, Katsikis I, Papadakis E, Andric Z, Panidis D (2016). Non-alcoholic fatty liver disease is associated with insulin resistance and lipid accumulation product in women with polycystic ovary syndrome. Hum Reprod.

[5] Vassilatou E, Vassiliadi DA, Salambasis K, Lazaridou H, Koutsomitopoulos N, Kelekis N, Kassanos D, Hadjidakis D, Dimitriadis G (2015). Increased prevalence of polycystic ovary syndrome in premenopausal women with nonalcoholic fatty liver disease. Eur J Endocrinol.

[6] Lizneva D, Suturina L, Walker W, Brakta S, Gavrilova-Jordan L, Azziz R (2016). Criteria, prevalence, and phenotypes of polycystic ovary syndrome. Fertil Steril.

[7] Rosenfield RL, Ehrmann DA (2016). The Pathogenesis of Polycystic Ovary Syndrome (PCOS): The Hypothesis of PCOS as Functional Ovarian Hyperandrogenism Revisited. Endocr Rev.

[8] Ma WL, Lai HC, Yeh S, Cai X, Chang C (2014). Androgen receptor roles in hepatocellular carcinoma, fatty liver, cirrhosis and hepatitis. Endocr Relat Cancer.

[9] Kanaya N, Vonderfecht S, Chen S (2013). Androgen (dihydrotestosterone)-mediated regulation of food intake and obesity in female mice. J Steroid Biochem Mol Biol.

[10] Lai H, Jia X, Yu Q, Zhang C, Qiao J, Guan Y, Kang J (2014). High-fat diet induces significant metabolic disorders in a mouse model of polycystic ovary syndrome. Biol Reprod.

[11] Navarro G, Allard C, Xu W, Mauvais-Jarvis F (2015). The role of androgens in metabolism, obesity, and diabetes in males and females. Obesity (Silver Spring).

[12] Tiniakos DG, Vos MB, Brunt EM (2010). Nonalcoholic fatty liver disease: pathology and pathogenesis. Annu Rev Pathol.

[13] Tilg H, Moschen AR (2008). Insulin resistance, inflammation, and non-alcoholic fatty liver disease. Trends Endocrinol Metab.

[14] Bogovich K, Clemons J, Poretsky L (1999). Insulin has a biphasic effect on the ability of human chorionic gonadotropin to induce ovarian cysts in the rat. Metabolism.

[15] Damario MA, Bogovich K, Liu HC, Rosenwaks Z, Poretsky L (2000). Synergistic effects of insulin-like growth factor-I and human chorionic gonadotropin in the rat ovary. Metabolism.

[16] Poretsky L, Clemons J, Bogovich K (1992). Hyperinsulinemia and human chorionic gonadotropin synergistically promote the growth of ovarian follicular cysts in rats. Metabolism.

[17] Kuscu NK, Koyuncu F, Ozbilgin K, Inan S, Tuglu I, Karaer O (2002). Insulin: does it induce follicular arrest in the rat ovary?. Gynecol Endocrinol.

[18] Lima MH, Souza LC, Caperuto LC, Bevilacqua E, Gasparetti AL, Zanuto R, Saad MJ, Carvalho CR (2006). Up-regulation of the phosphatidylinositol 3-kinase/protein kinase B pathway in the ovary of rats by chronic treatment with hCG and insulin. J Endocrinol.

[19] Zhang Y, Sun X, Sun X, Meng F, Hu M, Li X, Li W, Wu XK, Brännström M, Shao R, Billig H (2016). Molecular characterization of insulin resistance and glycolytic metabolism in the rat uterus. Sci Rep.

[20] Pawlak M, Lefebvre P, Staels B (2015). Molecular mechanism of PPARalpha action and its impact on lipid metabolism, inflammation and fibrosis in non-alcoholic fatty liver disease. J Hepatol.

[21] Guo S (2014). Insulin signaling, resistance, and the metabolic syndrome: insights from mouse models into disease mechanisms. J Endocrinol.

[22] Titchenell PM, Quinn WJ, Lu M, Chu Q, Lu W, Li C, Chen H, Monks BR, Chen J, Rabinowitz JD, Birnbaum MJ (2016). Direct Hepatocyte Insulin Signaling Is Required for Lipogenesis but Is Dispensable for the Suppression of Glucose Production. Cell Metab.

[23] Li Y, Soos TJ, Li X, Wu J, Degennaro M, Sun X, Littman DR, Birnbaum MJ, Polakiewicz RD (2004). Protein kinase C Theta inhibits insulin signaling by phosphorylating IRS1 at Ser(1101). J Biol Chem.

[24] Beurel E, Grieco SF, Jope RS (2015). Glycogen synthase kinase-3 (GSK3): regulation, actions, and diseases. Pharmacol Ther.

[25] Saltiel AR, Kahn CR (2001). Insulin signalling and the regulation of glucose and lipid metabolism. Nature.

[26] Schuppan D, Kim YO (2013). Evolving therapies for liver fibrosis. J Clin Invest.

[27] Browning JD, Horton JD (2004). Molecular mediators of hepatic steatosis and liver injury. J Clin Invest.

[28] Grant RW, Stephens JM (2015). Fat in flames: influence of cytokines and pattern recognition receptors on adipocyte lipolysis. Am J Physiol Endocrinol Metab.

[29] Mathis D (2013). Immunological goings-on in visceral adipose tissue. Cell Metab.

[30] Mizushima N, Yoshimori T, Levine B (2010). Methods in mammalian autophagy research. Cell.

[31] Clark JM, Brancati FL, Diehl AM (2003). The prevalence and etiology of elevated aminotransferase levels in the United States. Am J Gastroenterol.

[32] O'Byrne SM, Blaner WS (2013). Retinol and retinyl esters: biochemistry and physiology. J Lipid Res.

[33] Aigner E, Bachofner N, Klein K, De Geyter C, Hohla F, Patsch W, Datz C (2009). Retinol-binding protein 4 in polycystic ovary syndrome—association with steroid hormones and response to pioglitazone treatment. J Clin Endocrinol Metab.

[34] Mellati AA, Sharifi F, Sajadinejad M, Sohrabi D, Mazloomzadeh S (2012). The relationship between retinol-binding protein 4 levels, insulin resistance, androgen hormones and polycystic ovary syndrome. Scand J Clin Lab Invest.

[35] Sopher AB, Gerken AT, Blaner WS, Root JM, McMahon DJ, Oberfield SE (2012). Metabolic manifestations of polycystic ovary syndrome in nonobese adolescents: retinol-binding protein 4 and ectopic fat deposition. Fertil Steril.

[36] Mohlig M, Weickert MO, Ghadamgahi E, Arafat AM, Spranger J, Pfeiffer AF, Schofl C (2008). Retinol-binding protein 4 is associated with insulin resistance, but appears unsuited for metabolic screening in women with polycystic ovary syndrome. Eur J Endocrinol.

[37] Weiping L, Qingfeng C, Shikun M, Xiurong L, Hua Q, Xiaoshu B, Suhua Z, Qifu L (2006). Elevated serum RBP4 is associated with insulin resistance in women with polycystic ovary syndrome. Endocrine.

[38] Jones H, Sprung VS, Pugh CJ, Daousi C, Irwin A, Aziz N, Adams VL, Thomas EL, Bell JD, Kemp GJ, Cuthbertson DJ (2012). Polycystic ovary syndrome with hyperandrogenism is characterized by an increased risk of hepatic steatosis compared to nonhyperandrogenic PCOS phenotypes and healthy controls, independent of obesity and insulin resistance. J Clin Endocrinol Metab.

[39] Vassilatou E, Lafoyianni S, Vryonidou A, Ioannidis D, Kosma L, Katsoulis K, Papavassiliou E, Tzavara I (2010). Increased androgen bioavailability is associated with non-alcoholic fatty liver disease in women with polycystic ovary syndrome. Hum Reprod.

[40] Setji TL, Holland ND, Sanders LL, Pereira KC, Diehl AM, Brown AJ (2006). Nonalcoholic steatohepatitis and nonalcoholic Fatty liver disease in young women with polycystic ovary syndrome. J Clin Endocrinol Metab.

[41] Caldwell AS, Middleton LJ, Jimenez M, Desai R, McMahon AC, Allan CM, Handelsman DJ, Walters KA (2014). Characterization of reproductive, metabolic, and endocrine features of polycystic ovary syndrome in female hyperandrogenic mouse models. Endocrinology.

[42] Denechaud PD, Lopez-Mejia IC, Giralt A, Lai Q, Blanchet E, Delacuisine B, Nicolay BN, Dyson NJ, Bonner C, Pattou F, Annicotte JS, Fajas L (2016). E2F1 mediates sustained lipogenesis and contributes to hepatic steatosis. J Clin Invest.

[43] Bechmann LP, Hannivoort RA, Gerken G, Hotamisligil GS, Trauner M, Canbay A (2012). The interaction of hepatic lipid and glucose metabolism in liver diseases. J Hepatol.

[44] Tailleux A, Wouters K, Staels B (2012). Roles of PPARs in NAFLD: potential therapeutic targets. Biochim Biophys Acta.

[45] Paglialunga S, Dehn CA (2016). Clinical assessment of hepatic de novo lipogenesis in non-alcoholic fatty liver disease. Lipids Health Dis.

[46] Michael MD, Kulkarni RN, Postic C, Previs SF, Shulman GI, Magnuson MA, Kahn CR (2000). Loss of insulin signaling in hepatocytes leads to severe insulin resistance and progressive hepatic dysfunction. Mol Cell.

[47] Cerda C, Perez-Ayuso RM, Riquelme A, Soza A, Villaseca P, Sir-Petermann T, Espinoza M, Pizarro M, Solis N, Miquel JF, Arrese M (2007). Nonalcoholic fatty liver disease in women with polycystic ovary syndrome. J Hepatol.

[48] Gambarin-Gelwan M, Kinkhabwala SV, Schiano TD, Bodian C, Yeh HC, Futterweit W (2007). Prevalence of nonalcoholic fatty liver disease in women with polycystic ovary syndrome. Clin Gastroenterol Hepatol.

[49] Markou A, Androulakis II, Mourmouris C, Tsikkini A, Samara C, Sougioultzis S, Piaditis G, Kaltsas G (2010). Hepatic steatosis in young lean insulin resistant women with polycystic ovary syndrome. Fertil Steril.

[50] Seki E, Schwabe RF (2015). Hepatic inflammation and fibrosis: functional links and key pathways. Hepatology.

[51] Wang K (2015). Autophagy and apoptosis in liver injury. Cell Cycle.

[52] Madrigal-Matute J, Cuervo AM (2016). Regulation of Liver Metabolism by Autophagy. Gastroenterology.

[53] Liu HY, Han J, Cao SY, Hong T, Zhuo D, Shi J, Liu Z, Cao W (2009). Hepatic autophagy is suppressed in the presence of insulin resistance and hyperinsulinemia: inhibition of FoxO1-dependent expression of key autophagy genes by insulin. J Biol Chem.

[54] Kleiner DE, Brunt EM, Van Natta M, Behling C, Contos MJ, Cummings OW, Ferrell LD, Liu YC, Torbenson MS, Unalp-Arida A, Yeh M, McCullough AJ, Sanyal AJ, and Nonalcoholic Steatohepatitis Clinical Research Network (2005). Design and validation of a histological scoring system for nonalcoholic fatty liver disease. Hepatology.

[55] Mao Y, Yu F, Wang J, Guo C, Fan X (2016). Autophagy: a new target for nonalcoholic fatty liver disease therapy. Hepat Med.

[56] Shibata M, Yoshimura K, Tamura H, Ueno T, Nishimura T, Inoue T, Sasaki M, Koike M, Arai H, Kominami E, Uchiyama Y (2010). LC3, a microtubule-associated protein1A/B light chain3, is involved in cytoplasmic lipid droplet formation. Biochem Biophys Res Commun.

[57] D'Eon TM, Souza SC, Aronovitz M, Obin MS, Fried SK, Greenberg AS (2005). Estrogen regulation of adiposity and fuel partitioning. Evidence of genomic and non-genomic regulation of lipogenic and oxidative pathways. J Biol Chem.

[58] Jeong S, Yoon M (2007). Inhibition of the actions of peroxisome proliferator-activated receptor alpha on obesity by estrogen. Obesity (Silver Spring).

[59] Li X, Pishdari B, Cui P, Hu M, Yang HP, Guo YR, Jiang HY, Feng Y, Billig H, Shao R (2015). Regulation of androgen receptor expression alters AMPK phosphorylation in the endometrium: *in vivo* and *in vitro* studies in women with polycystic ovary syndrome. Int J Biol Sci.

[60] Matthews DR, Hosker JP, Rudenski AS, Naylor BA, Treacher DF, Turner RC (1985). Homeostasis model assessment: insulin resistance and beta-cell function from fasting plasma glucose and insulin concentrations in man. Diabetologia.

[61] DeUgarte CM, Bartolucci AA, Azziz R (2005). Prevalence of insulin resistance in the polycystic ovary syndrome using the homeostasis model assessment. Fertil Steril.

