# Supplementary material for: Hyperandrogenism and insulin resistance contribute to hepatic steatosis and inflammation in female rat liver
Source: Oncotarget. 2018 Feb 9;9(26):18180–97. doi: 10.18632/oncotarget.24477 (PMC5915065; doi:10.18632/oncotarget.24477)
Supplement: Supplementary file 1 [file oncotarget-09-18180-s001.pdf]

Hyperandrogenism and insulin resistance contribute to hepatic steatosis and inflammation in female rat liver

SUPPLEMENTARY MATERIALS

Supplementary Table 1: Antibodies: species, clone/catalog number, method, dilution, and source

Antibody	Species	Cat. No.	kDa	Method	Dilution	Source
IRα	Rabbit	07-724	97	WB	1:500	Millopore (Darmstadt, Germany)
IRβ	Rabbit	3025	95	WB	1:1000	Cell Signaling Technology (Danver, MA)
IRS1	Rabbit	3407	180	WB	1:500	Cell Signaling Technology
p-IRS1 (S1101)	Rabbit	2385	180	WB	1:300	Cell Signaling Technology
IRS2	Rabbit	4502	185	WB	1:200	Cell Signaling Technology
pan-Akt	Mouse	2920	60	WB	1:1000	Cell Signaling Technology
p-Akt (S473)	Rabbit	4060	60	WB	1:500	Cell Signaling Technology
p-Akt (T308)	Rabbit	13038	60	WB	1:500	Cell Signaling Technology
AS160	Rabbit	2670	160	WB	1:500	Cell Signaling Technology
GSK3α	Rabbit	4337	51	WB	1:2000	Cell Signaling Technology
p-GSK3α (S21)	Rabbit	9316	51	WB	1:500	Cell Signaling Technology
GSK3β	Rabbit	12456	46	WB	1:2000	Cell Signaling Technology
p-GSK3β (S9)	Rabbit	5558	46	WB	1:500	Cell Signaling Technology
Beclin-1	Rabbit	3495	60	WB	1:1000	Cell Signaling Technology
Atg7	Rabbit	8558	78	WB	1:1000	Cell Signaling Technology
Atg3	Rabbit	3415	40	WB	1:1000	Cell Signaling Technology
Atg5	Rabbit	12994	55	WB	1:1000	Cell Signaling Technology
Atg12	Rabbit	4180	16,53	WB	1:1000	Cell Signaling Technology
Atg16L1	Rabbit	8089	66,68	WB	1:1000	Cell Signaling Technology
LC3I/II	Rabbit	12741	14,16	WB	1:1000	Cell Signaling Technology
p62	Rabbit	5114	62	WB	1:1000	Cell Signaling Technology
α-SMA	Rabbit	6487	100	WB	1:1000	Cell Signaling Technology
AR	Rabbit	sc816	110	WB	1:100	Santa Cruz Biotechnology
IGF1R	Rabbit	9750	95	WB	1:1000	Cell Signaling Technology
p-IGF1R	Rabbit	3024	95	WB	1:500	Cell Signaling Technology
IGF2R	Rabbit	14364	275	WB	1:500	Cell Signaling Technology
IGFBP1	Rabbit	06-580	32	WB	1:500	Upstate (Darmstadt, Germany)
F4/80	Rabbit	ab74383	160	WB	1:1000	Abcam (Cambridge, UK)
MPO	Rabbit	sc16128	72	WB	1:100	Santa Cruz Biotechnology (Heidelberg, Germany)
CD4	Goat	sc1140	54	WB	1:100	Santa Cruz Biotechnology
TNFα	Goat	sc1349		IHC	1:150	Santa Cruz Biotechnology
IL1β	Rabbit	sc7884		IHC	1:100	Santa Cruz Biotechnology

IR, insulin receptor; IRS, insulin receptor substrate; AS160, the Akt Ser/Thr kinase 160; GSK3, glycogen synthase kinase 3; Atg, autophagy-related protein; LC3, microtubule-associated protein 1A/1B-light chain 3 (a cytosolic LC3-I is conjugated to phosphatidylethanolamine to form LC3-II, which is recruited to autophagosomal membranes); p62, sequestosome 1 (SQSTM1); α-SMA, α-smooth muscle actin; AR, androgen receptor; IGF1R, insulin-like growth factor receptor 1; IGFBP1, IGF binding protein 1; F4/80, adhesion G protein-coupled receptor E1; MPO, the heme protein myeloperoxidase; CD4, cluster of differentiation 4; TNFα, tumor necrosis factor α; IL1β, interleukin-1β; WB, western blot; IHC, immunohistochemistry.

Supplementery Table 2: Sequences of primer pairs used for for qRT-PCR measurement. See Supplementary_Table_2

Supplementary Table 3: The intra-assay and inter-assay % CV for the rat insulin, steroid hormones and lipid profile

	Intra-assay % CV	Inter-assay % CV
Insulin	6.4	7.5
E2	6.2	7.4
Total T	5.9	6.6
DHT	4.2	7.5
A4	6.0	7.4
RBP4	6.0	6.5
TC	2.1	4.0
TG	2.0	3.5
HDL-C	2.6	4.2
ALT	7.0	8.3
AST	7.2	8.1
PIIINP	6.2	7.3
HA	6.0	7.1

E2, 17β-estradiol; T, testosterone; DHT, dihydrotestosterone; A4, androstenedione; RBP4, retinol-binding protein 4; TC, total cholesterol; TG, triglyceride; HDL-C, high-density lipoprotein cholesterol; ALT, alanine aminotransferase; AST, aspartate aminotransferase; PIIINP, N-terminal propeptide of yype III collagen; HA, hyaluronic acid.

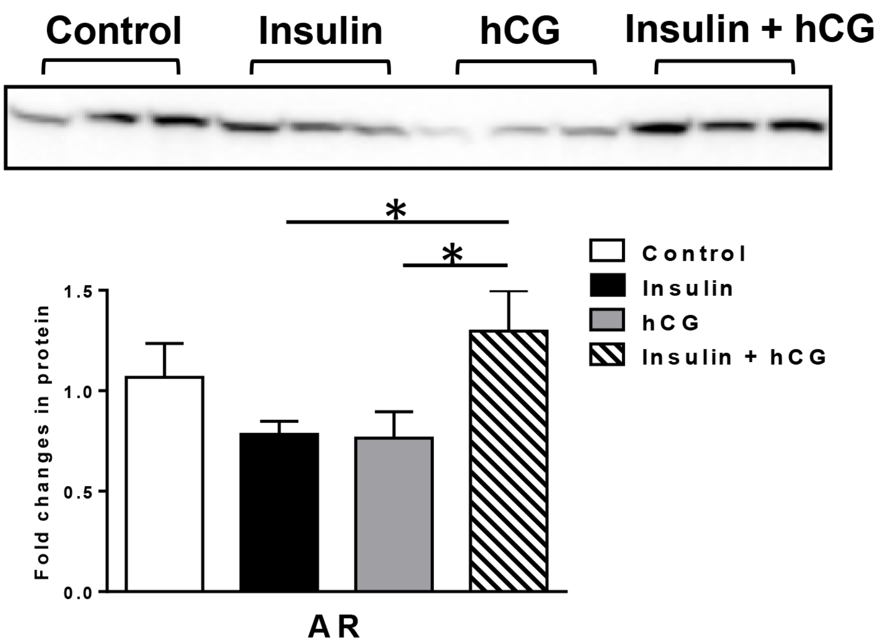

Supplementary Figure 1: Effects of insulin and/or hCG on the androgen receptor (AR) expression in the rat liver. Representative AR with immunoblotting quantification (n = 9/group) is shown, total proteins served as the loading control. Values are expressed as means ± SEM. $^{*}p < 0.05$.

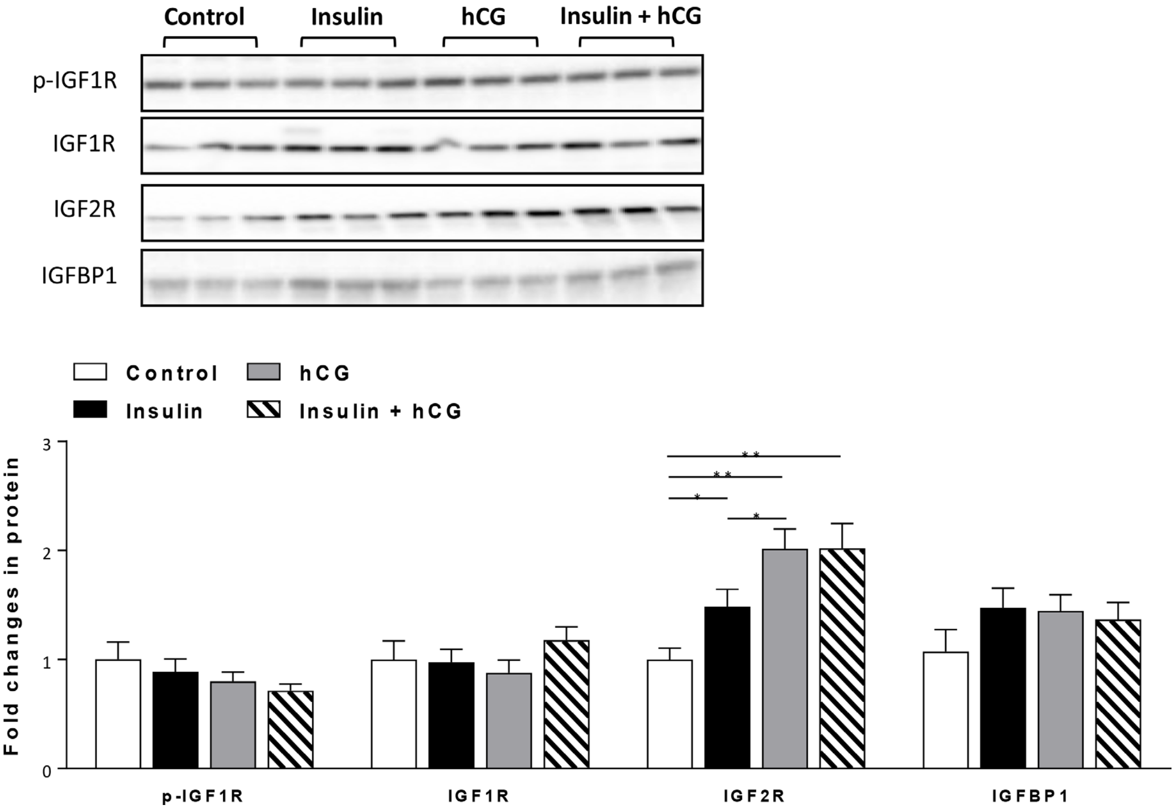

Supplementary Figure 2: Effects of insulin and/or hCG on insulin-like growth factor 1/2 receptor (IGF-1R and IGF2R) and IGF binding protein 1 (IGFBP1) expression in the rat liver. Representative p-IGF1R, IGF1R, IFG2R, and IGFBP1 are shown. For immunoblotting quantification (n = 9/group), total proteins served as the loading control. Values are expressed as means ± SEM. $^{*}p < 0.05$; $^{**}p < 0.01$.

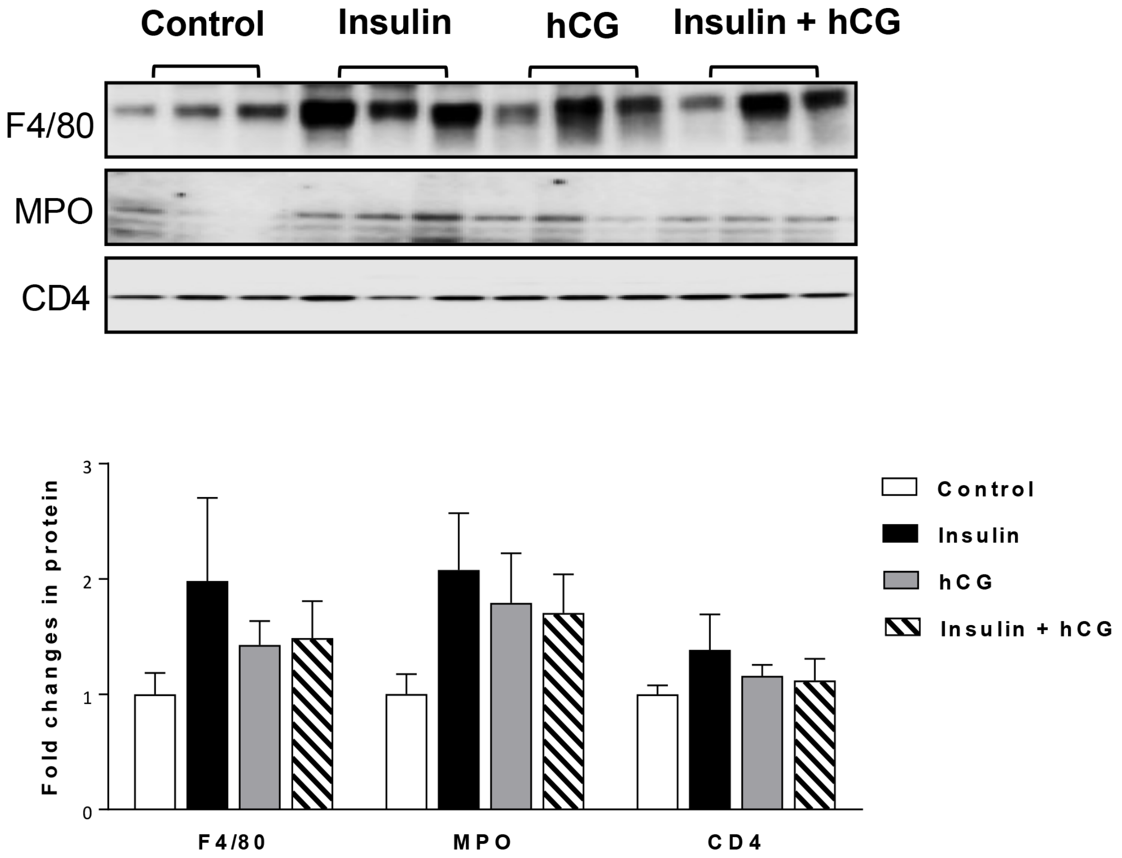

Supplementary Figure 3: Effects of insulin and/or hCG on F4/80, MPO, and CD4 expression in the rat liver. Representative F4/80, MPO, and CD4 are shown. For immunoblotting quantification (n = 9/group), total proteins served as the loading control. Values are expressed as means ± SEM.

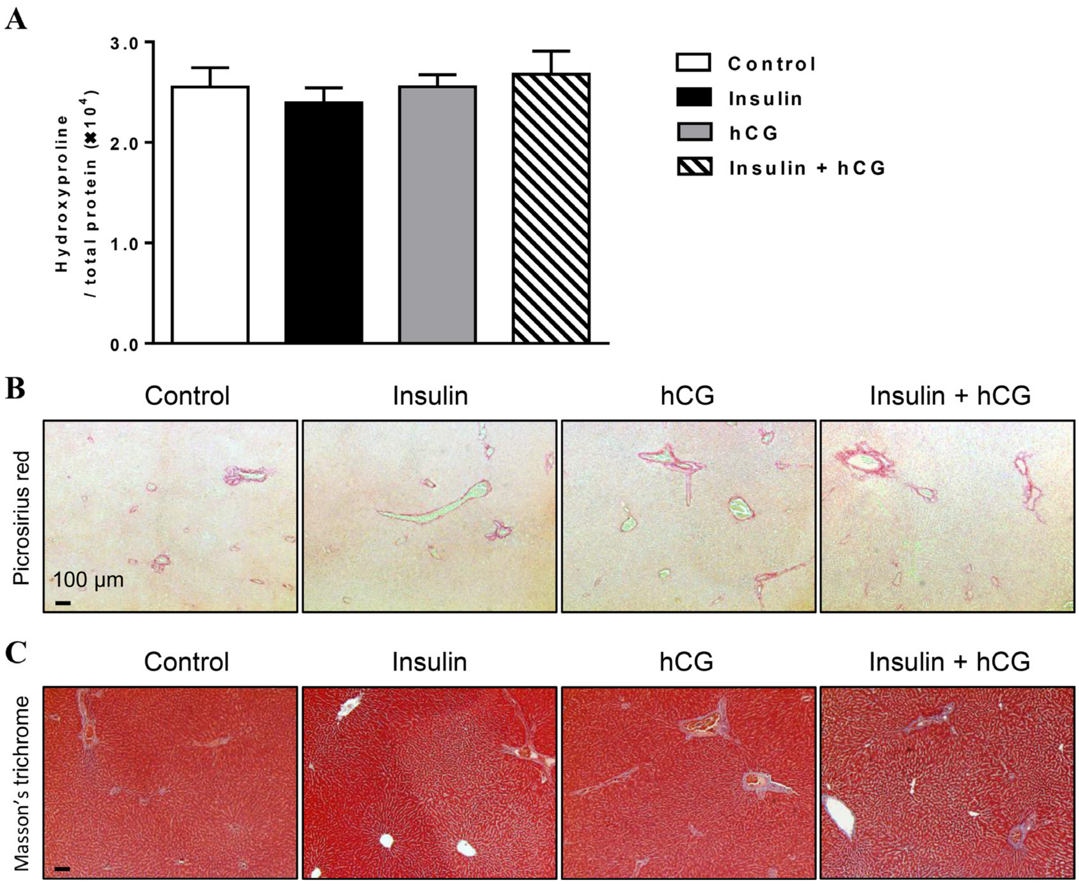

Supplementary Figure 4: Effects of insulin and/or hCG on collagen deposition in the rat liver. Picrosirius red (**A**) and Masson's trichrome (**B**) staining of liver tissue sections (n = 9–12/group) did not reveal any major histological differences in collagen deposition between groups regardless of different treatment. There was no significant difference in hepatic hydroxyproline levels (**C**) in any of the groups (n = 9/group). Scale bars are indicated in the photomicrographs.
