# Supplementary material for: Hyperandrogenism and insulin resistance contribute to hepatic steatosis and inflammation in female rat liver
Source: Oncotarget. 2018 Feb 9;9(26):18180–97. doi: 10.18632/oncotarget.24477 (PMC5915065; doi:10.18632/oncotarget.24477)
Supplement: Supplementary file 2 [file oncotarget-09-18180-s002.docx]

**Supplemented Table 2: Sequences of primer pairs used for for qRT-PCR measurement**

| **Gene** |  | **Primer** | **Size** |
| --- | --- | --- | --- |
| *E2f1* | Forward | ATCCTGACGTGCTGCTCTTCG | 114 bp |
|  | Reverse | GGTCAGTTTCCAGATCCAGCC |  |
| *Pparg* | Forward | TGAAGACATCCCGTTCACAAG | 100 bp |
|  | Reverse | CGCAGGCTCTACTTTGATCG |  |
| *Scd1* | Forward | TGTGGGGCGGTTATTTGTGA | 130bp |
|  | Reverse | CAGGCAGCACAGCTAGATCC |  |
| *Gpam* | Forward | TGAAAGCTGCAACTGAGACG | 112 bp |
|  | Reverse | GGAGCTTTGATGTTGTGGCA |  |
| *Ppara* | Forward | GGCTCTGAACATTGGCGTTC | 96 bp |
|  | Reverse | CAAGGGGACAACCAGAGGAC |  |
| *Cpt1α* | Forward | ACAATGGGACATTCCAGGAG | 3216 bp |
|  | Reverse | AAAGACTGGCGCTGCTCA |  |
| *Ucp2* | Forward | GCAGTTCTACACCAAGGGCT | 122bp |
|  | Reverse | GGAAGCGGACCTTTACCACA |  |
| *Lxrα* | Forward | GAGGGCTGCAAGGGATTCTT | 128 bp |
|  | Reverse | CATTTGCGAAGGCGACACTC |  |
| *Srebp2* | Forward | TTGACTCAGACAGCCAATGG | 139 bp |
|  | Reverse | GCGTGGTCAAAACAAGGGAA |  |
| *Srebp1* | Forward | GGACGAGCTACCCTTCGGT | 167 bp |
|  | Reverse | CTGTCTCACCCCCAGCATAG |  |
| *Acc1* | Forward | CACATCATGAAGGAGGAGG | 276 bp |
|  | Reverse | GCTATCACACAGCCTGGGTC |  |
| *Ehhadh* | Forward | TTCTGGATGCAGTCGTGAAG | 86 bp |
|  | Reverse | GCGGGGTTCTATGGGTTTAT |  |
| *Fgf21* | Forward | GCCAGGGGTCATTCAAATCC | 157 bp |
|  | Reverse | GGCCTCAGACTGGTACACAT |  |
| *Acox1* | Forward | CTGATGAAATACGCCCAGGT | 204 bp |
|  | Reverse | CTGTGGTTCTGGTTCGCTTT |  |
| **Gene** |  | **Primer** | **Size** |
| *Hmgcs* | Forward | GCCTACAGGTGGAGTTGGAG | 216 bp |
|  | Reverse | CGGATCTTTTTGCGGTAGAC |  |
| *Il6* | Forward | CTGGTCTTCTGGAGTTCCGT | 219 bp |
|  | Reverse | TGGTCCTTAGCCACTCCTTCT |  |
| *Mcp1* | Forward | GGGGTCTTGCAGTATTGGC | 119 bp |
|  | Reverse | ACGCTTCCAGCTGTCTTCTTC |  |
| *Bcl2* | Forward | GGATACTGGAGATGAAGACT | 567 bp |
|  | Reverse | GCTGAGCAGCGTCTTCAGAG |  |
| *Bax* | Forward | GGAGGAAGTCCAGTGTCCAG | 207 bp |
|  | Reverse | TGCAGAGGATGATTGCTGAC |  |
| *Caspase3* | Forward | CAGCTCGCAATGGTACCGAT | 620 bp |
|  | Reverse | GCATTGACACAATACACGGG |  |
| *Tgfb* | Forward | CACTCCCGTGGCTTCTAGTG | 145 bp |
|  | Reverse | GGACTGGCGAGCCTTAGTTT |  |
| *Ctgf* | Forward | TCTCTGCTCCTCCCTGTTCTA | 121 bp |
|  | Reverse | GGTAACCAGGCGTCCGATAC |  |
| *Rbp4* | Forward | AAGGGACGAGTCCGTCTTCT | 182 bp |
|  | Reverse | TACTGCAGAGCGAAGGTGTC |  |
| *F4/80* | Forward | CTACAGCTGTTCTTGCCCAG | 123 bp |
|  | Reverse | CATGATGAGTTGGAAGGGCA |  |
| *Cd11c* | Forward | GGGACAGGTTGGACTATGGT | 112 bp |
|  | Reverse | AATTCTTGCCTGGACTGTGC |  |
| *Il6 (Adipose tissue)* | Forward | AGTTCCGTTTCTACCTGGAGTT | 163 bp |
|  | Reverse | AGAGCATTGGAAGTTGGGGT |  |
| *Mip1α* | Forward | GGACGGCAAATTCCACGAAA | 126 bp |
|  | Reverse | CCAGGTCTCTTTGGGGTCAG |  |
| *Il1β* | Forward | AGGAGAGACAAGCAACGACA | 238 bp |
|  | Reverse | TGCTTGAGAGGTGCTGATGT |  |
| **Gene** |  | **Primer** | **Size** |
| *Il1rα* | Forward | CGCTTTACCTTCATCCGCTC | 131 bp |
|  | Reverse | GGCTCTTTTGGTGTGTTGGT |  |
| *Gapdh* | Forward | TGACAACTTTGGCATCGTGG | 78 bp |
|  | Reverse | GGGCCATCCACAGTCTTCTG |  |
| *U87* | Forward | CCAGGTGCAACAAAACCTGT | 188 bp |
|  | Reverse | GCTGGACCCAAAACAACGAG |  |

*E2f1,* E2F transcription factor 1; *Pparg,* peroxisome proliferator activated receptor gamma; *Scd1,* stearoyl-coenzyme A (CoA) desaturase-1; *Gpam,* mitochondrial glycerol-3-phosphate acyltransferase; *Cpt1α,* carnitine palmitoyltransferase 1 alpha; *Ucp2,* uncoupling protein 2; *Lxrα,* liver X receptor alpha; *Srebp1,* sterol regulatory element-binding protein 1; *Acc1*, acetyl-CoA carboxylase 1; *Ppara,* peroxisome proliferator activated receptor alpha; *Ehhadh*, enoyl-CoA hydratase and 3-hydroxyacyl CoA dehydrogenase; *Fgf21*, fibroblast growth factor 21; *Acox1*, acyl-CoA oxidase 1; *Hmgcs*, hydroxymethylglutaryl-CoA synthase; *Il6,* interleukin 6; *Mcp1,* monocyte chemotactic protein 1 (*Ccl2*); *Bcl2,* B-cell lymphoma 2; *Bax*, bcl2-associated x protein; *Tgfb,* transforming growth factor beta; *Ctgf,* connective tissue growth factor; *Rbp4,* Retinol binding protein 4; *F4/80,* adhesion G protein-coupled receptor E1 (*Adgre1*); *Cd11c,* integrin alpha X (*Itgax*); *Mip1α*, macrophage inflammatory protein 1 alpha (Ccl3); *Gapdh,* glyceraldehyde-3-phosphate dehydrogenase; *U87,* small nucleolar RNA, C/D box 87 *(Snord87)*.
